# Supplementary material for: Vibronically assisted sub-cycle charge transfer at a non-fullerene acceptor heterojunction
Source: Nat Commun. 2026 Mar 5;17:2165. doi: 10.1038/s41467-026-70292-8 (PMC12963379; doi:10.1038/s41467-026-70292-8)
Supplement: Supplementary file 1 — Supplementary Information [file 41467_2026_70292_MOESM1_ESM.pdf]

Supplementary Information for

Vibronically Assisted Sub-Cycle Charge Transfer at a Non-Fullerene Acceptor Heterojunction

Pratyush Ghosh[1], Jeroen Royakkers[2], Giacomo Londi[3], Samuele Giannini[3], Rakesh Arul[1], Alexander J. Gillett[1,4], Scott T. Keene[1,5], Szymon J. Zelewski[1,6], David Beljonne[7*], Hugo Bronstein[1,2*], Akshay Rao[1*]

Affiliation:

[1]Cavendish Laboratory, University of Cambridge, Cambridge, UK

[2]Yusuf Hamied Department of Chemistry, University of Cambridge, Cambridge, UK

[3]Department of Chemistry and Industrial Chemistry, University of Pisa, 56124 Pisa, Italy

[4]Department of Physics, Chemistry and Biology (IFM), Linköping University, 581 83 Linköping, Sweden

[5]Department of Materials Science and NanoEngineering, Rice University, Houston, TX 77005, USA

[6]Department of Experimental Physics, Wroclaw University of Science and Technology, 50-370 Wroclaw, Poland

[7]Laboratory for Chemistry of Novel Materials, University of Mons, Mons, Belgium

corresponding authors (*):

Akshay Rao: ar525@cam.ac.uk

Hugo Bronstein: hab60@cam.ac.uk

David Beljonne: David.BELJONNE@umons.ac.be

Table of Contents

		Page #
SI Note 1	Energy Resolved–Electrochemical Impedance Spectroscopy (ER-EIS) of the Reference polymer Ref-P	3
SI Note 2	Calculated charge-transfer transitions are off-resonant to the impulsive red pump	3
SI Note 3	Characterization of the Ref-P hole polaron (h+) signature	5
SI Note 4	Spectral and temporal signature of the impulsive pumps and the effective time resolution of the processes	6
SI Note 5	Spectral characterization of the PDI radical anion	8
SI Note 6	Diluted space polymers	9
SI Note 7	Wavelength-resolved dynamics of the sub-vibrational period electron transfer	11
SI Note 8	Picosecond transient absorption spectroscopy with the IR- probe	16
SI Note 9	~200 fs hole transfer dynamics from the PDI to the polymer backbone	17
SI Note 10	(Pre)Resonant Impulsive vibrational spectroscopy of the PDI with an resonant probe	20
SI Note 11	Resonant Impulsive vibrational spectroscopy of the chemically reduced PDI radical anion with an resonant probe	21
SI Note 12	Off-resonant Impulsive vibrational spectroscopy of the PDI	22
SI Note 13	Additional Evidence Against Direct Excitation of PDI by Red-Shifted Impulsive Pump	25
SI Note 14	Description of the anharmonicity in the impulsive excitation	26
SI Note 15	Quantum chemical calculations	31
SI Note 16	Microscopic Origin of Charge Transfer	38
SI Note 17	Extraction of the ultrafast charge-transfer dynamics in model heterojunction	40
SI Note 18	Further discussion on the assignment of the 1283 cm^{-1} Vibrational Coherence in TS-P3	42
SI Note 19	Additional cw-Raman Analysis of PDI and TS-P3 Derivatives	45
SI Note 20	Narrowband filtered inverse FFT and short time FFT (STFT) analysis	47
SI Note 21	Excited-state Quantum dynamics	49

[1]Supplementary Note 1: Energy Resolved–Electrochemical Impedance Spectroscopy (ER-EIS) of the Reference polymer Ref-P.

The Density of states (DOS) functions[1] of the HOMO and LUMO states of the neat film of the Ref-P (in the micro-device architecture described in method section) are shown in the Supplementary Fig. S1. The HOMO and LUMO energies are listed in the Supplementary Table 1.

Supplementary Table S1 | ER-EIS measurement fitting parameters[1]

	E_0 [eV]	σ[meV]	E_{edge}[eV]	Scale (a.u.)
**LUMO**	-3.49	60	-3.73	0.0000189
**HOMO**	-5.69	105	-5.25	0.0000289

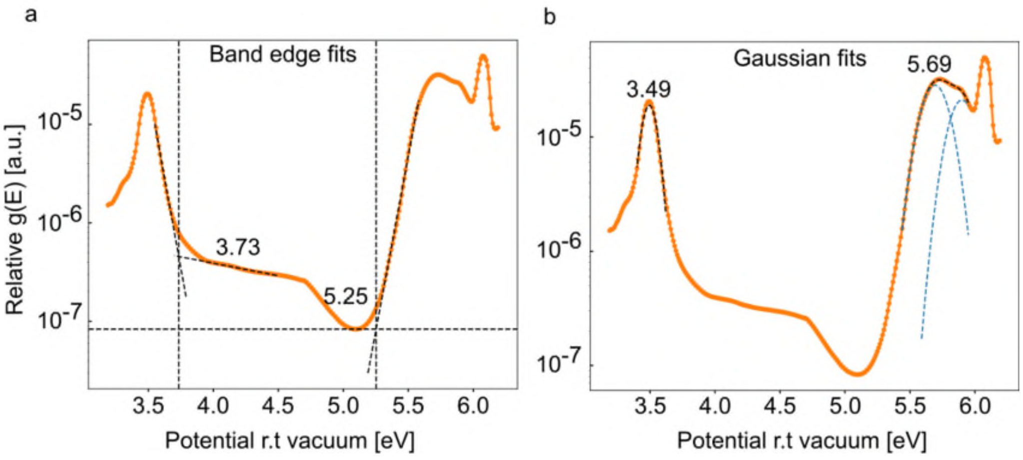

Supplementary Fig. 1| Density of states function g(E) for the HOMO and LUMO states for the neat film of the Ref-P: a, fits for the band edge, b, Gaussian fits;

Supplementary Note 2: Calculated charge-transfer transitions are off-resonant to the impulsive red pump

It has been shown that TS-P2 based devices exhibit an order of magnitude higher electroluminescence (EL) compared to TS-P3 under the same injection current[2]. This is attributed to the fact that the oscillator strength of the charge-transfer (CT) transitions is nearly an order of magnitude higher in TS-P2 than in TS-P3[2]. Additionally, the CT transitions in TS-P2 are closer in energy to the excitonic peak, allowing for an 'energy-borrowing' effect[2].

[1] The fitting function used in the fitting of the ER-EIS data is

$$y = scale * e^{-0.5\left(\frac{x-E_0}{\sigma}\right)^2} \quad \text{(S1)}$$

Consequently, the charge-transfer transitions are less dark in TS-P2 compared to TS-P3. In the TS-P3 system, the calculated highest energy charge-transfer state has 3,600 times weaker oscillator strength than the excitonic transition of the polymer backbone chromophore, and it is 144 nm red-shifted[2], making it clearly off-resonant from the pump spectra used to impulsively excite the backbone chromophore (Supplementary Fig. 2). This further supports that the PDI radical anion and the polymer hole polaron signals we observed are outcomes of rapid excited-state charge transfer rather than direct excitation of the charge-transfer transition.

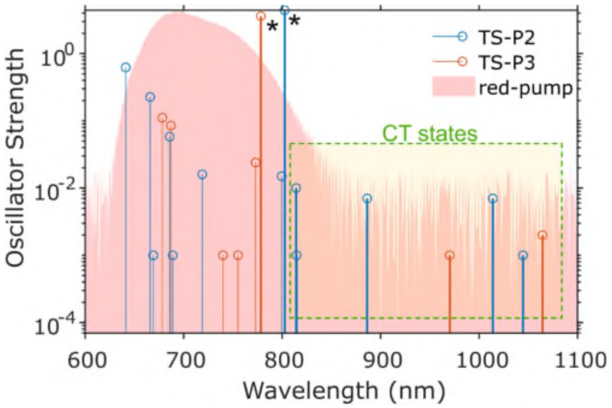

Supplementary Fig. 2| Simulated absorption spectra of the TS-P2 and TS-P3. The data is taken from Royakkers *et al*[2]. The asterisk(*) transitions are the lowest energy excitonic state and the transitions that are inside the green box are the charge-transfer transitions. The red filled area (used in our experiment) represents the red pump used to impulsively photoexcite the polymer backbone chromophore.

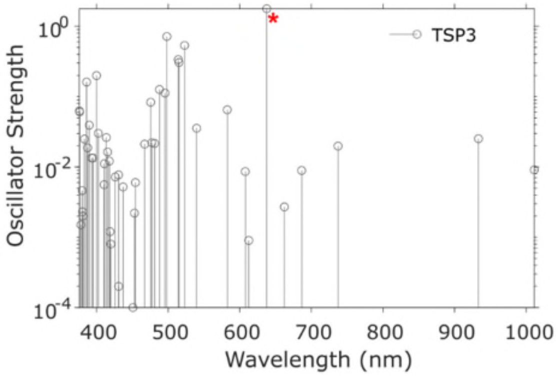

Supplementary Fig. 3| Calculated electronic transitions of the DPP-BDT dimer of the TS-P3 architecture. Asterisked mode corresponds to the Frenkel exciton (FE). excited-state calculations is performed using 6-311G(d,p) basis set with range-separated hybrid functional (specifically designed for the characterization of long-range effects as CT states) which further reveal oscillator strengths of the charge-transfer (CT) transitions are roughly 800 times weaker than those of the lowest asterisked Frenkel excitonic transitions.

Supplementary Note 3: Characterization of the Ref-P hole polaron (h^+) signature

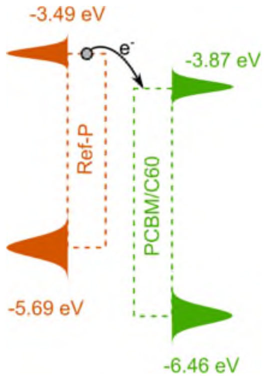

Supplementary Fig. 4| Energy level diagram of the Ref-P and PCBM/C60 obtained from the energy-resolved-Electrochemical impedance spectroscopy (for technique see method and reference[1]). The data for the PCBM/C60 is obtained from the reference[1].

As shown in Supplementary Fig. 4, the energy level diagram of Ref-P and PCBM/C60 indicates a 380 meV offset between their LUMO levels. The pump spectra (Supplementary Fig. 5a) used to excite the polymer chromophore is completely off-resonant with the PCBM/C60 absorption[3]. Therefore, we can safely exclude the possibility of energy transfer or hole transfer mechanisms as the cause for the new band formed around 1300 nm upon adding PCBM/C60. Instead, this new band is attributed to electron transfer from the Ref-P polymer to PCBM/C60, as supported by the energy level diagram (Supplementary Fig. 4).

The hole polaronic feature observed in the TS-P3 as a result of ultrafast electron transfer to PDI after photoexciting the polymer backbone is 80 meV blue-shifted compared to the spectra presented in Supplementary Fig. 5. The small spectral shift can be attributed to differences in the delocalization length of the hole polaron[4]. The delocalization length varies when comparing inter-chain interactions in solution to intra-chain interactions in film. Hole polaronic feature

for the similar BDT-DPP based conjugated polymers as such copolymers are observed in the very similar spectra regime has been observed earlier[5].

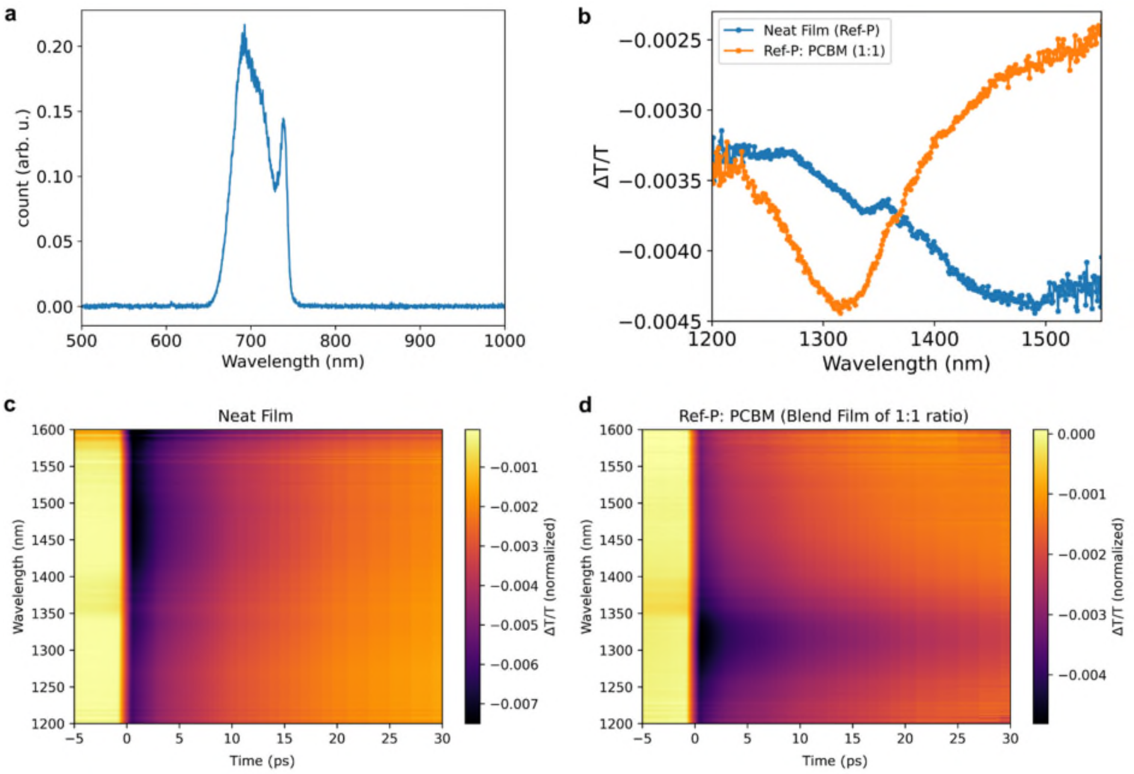

Supplementary Fig. 5| Characterization of the hole polaron (h^+) of the polymer backbone chromophore: a, spectral profile of the pump spectra used in the experiment. **b,** Differential transient absorption (ΔT/T) spectra averaged from the time delay of 5-15 ps plotted for the neat film of Ref-P (blue) and blended film of the Ref-P with PCBM/C60 in 1:1 wt% ratio. **c,d,** The spectrally and time resolved transient absorption map of the c, neat film of the Ref-P polymer and d, 1:1 wt% blend of the Ref-P and PCBM/C60.

Supplementary Note 4: Spectral and temporal signature of the impulsive pumps and the effective time resolution of the processes

As shown in the Supplementary Fig. 6, the time resolution of the red impulsive pump retrieved from the Frequency resolved optical gating (SHG-FROG) is 11.8 fs. In this scenario, the effective pulse duration of the probe pulse is replaced by its transform limit (TL), provided that the spectrometer used has a sufficiently high spectral resolution[6]. Consequently, for a 1030 nm white-light (WL) probe with a TL of 4 fs, the effective time resolution becomes limited by the pump pulse.

The effective time resolution can be determined from the variance (σ^2) of the convolution of the two Gaussian functions representing the pump and probe pulses as follows

$$\sigma = \left(\sigma_{pump}^2 + \sigma_{probe}^2\right)^{\frac{1}{2}} = \sqrt{(11.8^2 + 4^2)} = 12.45\text{fs}(\text{S2})$$

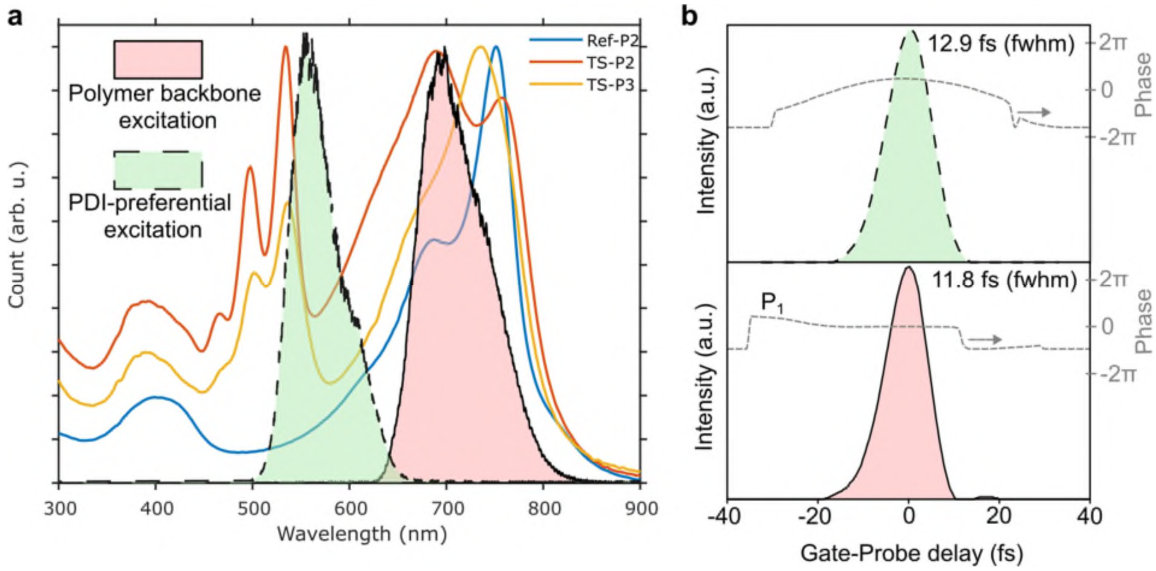

Supplementary Fig. 6| spectral and temporal profile of the broadband ultrafast pulses (sub 12 fs) used in the experiment: a, Absorption spectra of the Ref-P (blue curve), TS-P2(red curve), TS-P3 (yellow curve). The green filled (with dotted black line) area shows the pump used for the predominant PDI excitation. The red filled (with solid black line) represent the red impulsive pump. b, The FROG-retrieved temporal traces of the impulsive pumps used in the study

We note the electron transfer from the polymer backbone to the PDI is happening in the timescale of sub-15 fs. As the neutral state to the charge transfer state formation is exponential decay of the neutral state, the overall convoluted function can be represented as an exponentially modified Gaussian (EMG)[7] function with the functional form represented as[7] :

$$f(t) = \frac{\lambda}{2} exp^{\left(\frac{\lambda}{2}(2\mu + \lambda\sigma^2 - 2t)\right)} erfc\left(\frac{\mu + \lambda\sigma^2 - t}{\sqrt{2}c}\right)(\text{S3})$$

Where μ, σ^2 are mean and standard deviation of the convolved gaussian of the pump-probe experiment. λ is the decay constant of the neutral ref-P to the charge transfer state of the exponential (with mean lifetime $\frac{1}{\lambda}$).

The time resolution of the overall process is $\sqrt{\sigma^2 + \frac{1}{\lambda^2}}$, which is <$\sqrt{12.45^{\ 2} + 15^2}$ yielding a value of less than 19.4 fs.

Supplementary Note 5: Spectral characterization of the PDI radical anion

In Supplementary Fig. 7, the differential transmission signal (ΔT/T) for the TS-P3 system is presented at 20 fs and 40 fs, represented by the solid dark blue and green curves, respectively. As noted in Fig. 2d (main text), the Ref-P system exhibits no dynamics on this ultrafast timescale. Therefore, the difference Δ(ΔT/T) = ΔT/T(20 fs) - ΔT/T(40 fs) should predominantly reflect the absorption of the PDI radical anion, provided there is minimal spectral contribution from other features, such as the hole polaron associated with the polymer backbone.

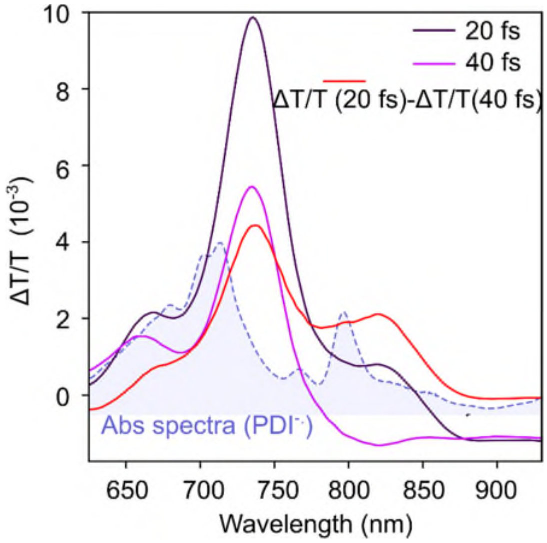

Supplementary Fig. 7| Spectral characterization of the PDI radical anion: The blue shaded are with dotted line corresponds to the absorption spectra of chemically reduced PDI radical anion.

The red spectrum for Δ(ΔT/T) closely aligns with the characteristic absorption of the PDI radical anion, reinforcing the identification of this process as ultrafast electron transfer. A slight red shift in the Δ(ΔT/T) spectrum relative to the PDI radical anion absorption may arise from variations in dielectric stabilization: while the PDI radical anion in solution is freely solvated, in the TS-P3 system it remains strongly bound to the hole polaron on the polymer backbone.

Supplementary Note 6: Diluted space polymers

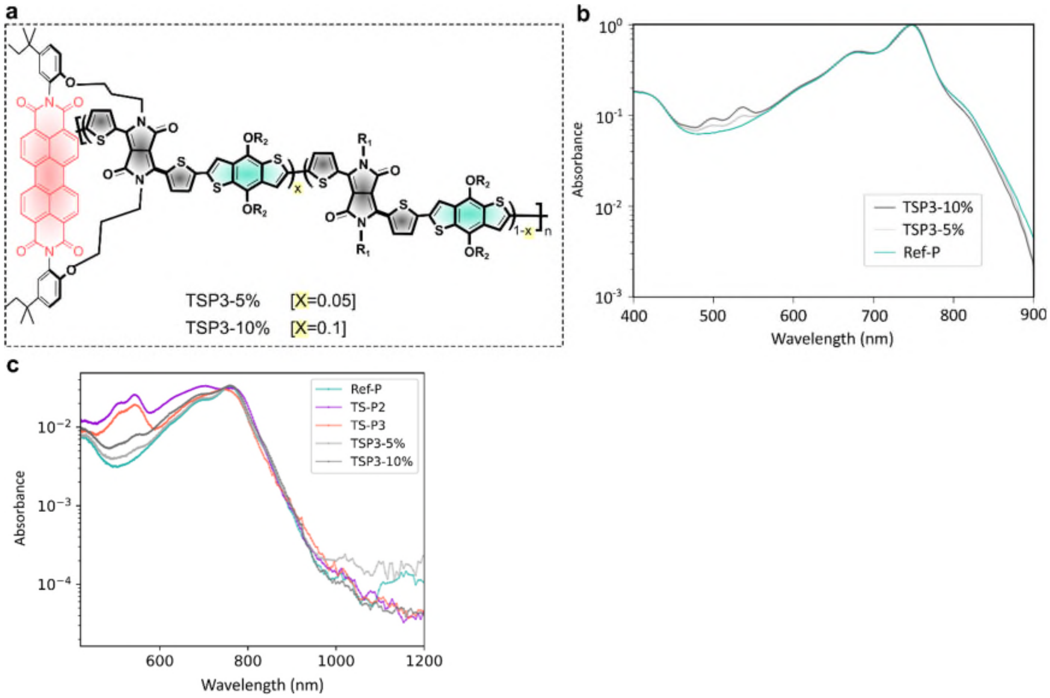

Supplementary Fig. 8 | Structural and spectroscopic characterization of diluted space polymers: a, Chemical structures of the diluted space polymers TSP3-5% and TSP3-10%, showing reduced density of the through-space PDI units relative to TS-P3. **b,** Normalized UV-Vis absorption spectra of TSP3-5% and TSP3-10%, highlighting the backbone-dominated spectral features and the absence of strong ground-state electronic coupling. **c,** Photothermal deflection spectra (PDS) of the diluted polymers, showing no low-energy absorption tails, further confirming the decoupling of PDI units from the polymer backbone in the ground state.

We have also synthesized and investigated a series of diluted-space polymers (TSP3-5% and TSP3-10%), which maintain a structural resemblance to the original TS-P3 polymer but feature a reduced number of PDI units per BDT-DPP backbone as shown in Supplementary Fig. 8a. This strategic dilution alleviates steric strain[2] on the polymer backbone, resulting in absorption spectra that more clearly reflect the intrinsic characteristics of the BDT-DPP chromophore. Notably, in the longer wavelength region, the absorption profiles of these diluted polymers (Supplementary Fig. 8b) closely match that of the backbone, indicating the absence of strong ground-state electronic coupling. This conclusion is further supported by photo-thermal absorption spectra (Supplementary Fig. 8c), which show no signs of low-energy features typically associated with such coupling.

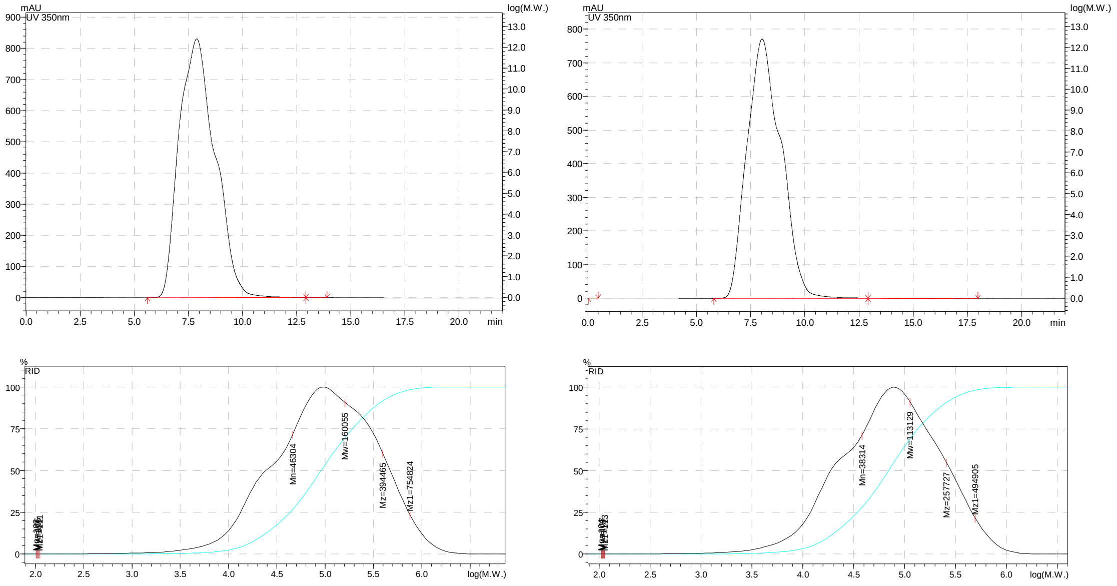

Supplementary Fig. 9 | GPC chromatograms of the diluted space polymers: GPC traces of TSP3-5% (left) and TSP3-10% (right), recorded using UV detection (top panels) and refractive index detection (RID, bottom panels). The chromatograms confirm successful polymer formation with distinct molecular weight distributions and no significant low-molecular-weight impurities.

Polymer Synthesis and Composition: All polymers were synthesized following previously established protocols[2]. In the diluted-space polymer variants, TSP3-5% and TSP3-10%, the molar feed ratios of DT-BDT-$SnMe_3$ to Br_2Th_2-DPP-2OD to the through-space monomer 3 (TSM3) were adjusted to 1:0.95:0.05 and 1:0.9:0.1, respectively.

Synthesis of TSP3-5%: Under an argon atmosphere, a solution of dry toluene (1.35 mL) and dry DMF (0.12 mL) was added to a mixture of DT-BDT-$SnMe_3$ (80.0 mg, 0.0655 mmol), Br_2Th_2-DPP-2OD (63.4 mg, 0.0622 mmol), TSM3 (4.1 mg, 0.0032 mmol), and $Pd(PPh_3)_4$ (1.7 mg, 0.0015 mmol). The reaction mixture was stirred at 110 °C for 12 hours. Upon cooling, it was precipitated into stirring methanol (~100 mL). The crude polymer was collected by filtration and sequentially purified via Soxhlet extraction with acetone (5 hours reflux) followed by hexane. The final hexane fraction was reprecipitated into methanol and filtered to yield 108 mg of the target polymer.

M_n = 46.3 kDa, $Đ$ = 2.16

Synthesis of TSP3-10%: A similar procedure was followed for TSP3-10%, with a reaction mixture comprising DT-BDT-$SnMe_3$ (80.0 mg, 0.0655 mmol), Br_2Th_2-DPP-2OD (60.1 mg, 0.0590 mmol), TSM3 (4.1 mg, 0.0065 mmol), and $Pd(PPh_3)_4$ (1.7 mg, 0.0015 mmol) in dry toluene (1.35 mL) and DMF (0.12 mL). Stirring at 110 °C was maintained for 12 hours under argon. The polymer was precipitated into methanol, filtered, and purified using Soxhlet

extraction (acetone followed by hexane). Final reprecipitation from hexane into methanol afforded 102 mg of purified polymer.

M_n = 38.3 kDa, *Đ* = 2.95

Supplementary Note 7: Additional discussion on the vibronic coupling of the model heterojunction

Here we discussed the vibronic coupling of model heterojunctions. In the electronic excited state (S_1) manifold, the vibrational ground state is represented as $\psi_{i=0}(x)$ and the first excited vibrational states are $\psi_{i=1}(x)$. After impulsive photoexcitation by a broadband laser pulse from the electronic ground state, multiple vibrational states will be populated, leading to, a newly generated non-stationary states which can be represented as:

$$\psi(r,t) = c_{i=0}(t)e^{-i\omega_0 t}\psi_{i=0}(r) + c_{i=1}(t)e^{-i\omega_1 t}\psi_{i=1}(r) + \cdots \quad \text{(S4)}$$

Where $c_{i=0}$, $c_{i=1}$, corresponds to the contribution of each vibrational states to the non-eigen state.

The time-dependent molecular polarization can be described as -

$$P(t) = \langle \psi(r,t)|\mu|\psi(r,t)\rangle \qquad (S5)$$

Where μ is the dipole moment operator.

$$P(t) = \left\langle c_{i=0}(t)e^{-i\omega_0 t}\psi_{i=0}(r) + c_{i=1}(t)e^{-i\omega_1 t}\psi_{i=1}(r)\middle|\mu\middle|c_{i=0}(t)e^{-i\omega_0 t}\psi_{i=0}(r) + c_{i=1}(t)e^{-i\omega_1 t}\psi_{i=1}(r)\right\rangle$$

$$P(t) = \mu_{01}(c_0^* c_1 e^{-i(\omega_1-\omega_0)t} + c_1^* c_0 e^{i(\omega_1-\omega_0)t}) \qquad \text{(S6)}$$

The macroscopic polarization can be represented as $P_N(t) = N.P(t)$, where N is the number of molecules[8]. Hence, the vibrational coherence generated by the superposition of two vibrational states can oscillate with $(\omega_1 - \omega_0) = \omega^{|es\rangle}$. The oscillatory time dependent change in the macroscopic polarization will vanish if the energetic bandwidth of the excitation laser source is lower than $\omega^{|es\rangle}$.

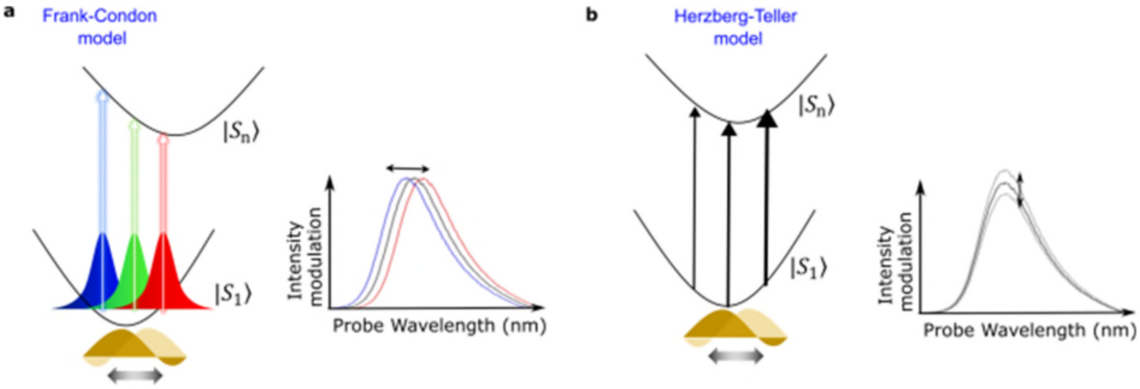

Supplementary Fig. 10 | Two kind of resonant probing mechanism: a, Frank-condon model and b, non-Condon Herzberg Teller model

Vibrational coherence can be detected through two complementary mechanisms. In the Franck–Condon (FC) picture (supplementary fig. 10a), nuclear motion periodically displaces the excited-state potential energy surface, leading to periodic red–blue shifts of electronic transitions in the transient absorption spectra. This results in a characteristic modulation of peak positions, which is readily observed when spectral features are well isolated. In contrast, in the Herzberg–Teller (HT) mechanism[9–11], vibrational motion modulates the electronic transition dipole moment or interstate coupling, producing oscillatory intensity changes in the transient features rather than pronounced spectral shifts. As a result of that if the vibrational coherence detection mechanism is not HT type mechanism then it is possible to detect the oscillatory spectral movement is possible.

Nuclear wavepacket motion in the vibration co-ordinate can be used as a probe for exciton-vibrational coupling[12]. In such impulsive excitation, the amount of the oscillation is dependent on the electron−phonon coupling strengths are represented by a set of parameters [g, S, λ]. g is a dimensionless displacement of the normal coordinate (as discussed above), S is the Huang−Rhys parameter, and E^{rel} is the reorganization energy.

Both S and λ exclusively dependent on the displacement (Δ) as follows:

$$S = \frac{g^2}{2}; \qquad E^{rel} = \hbar\omega S \qquad (S7)$$

where ω is the frequency of the optical phonon. E^{rel} ($\hbar\Delta\omega$) can be calculated from impulsive vibrational spectroscopy data using $A_{OSC} = \left(\frac{d\,OD}{d\omega}\right)\Delta\omega$, where OD is the optical density of the sample and A_{OSC} is the amplitude of the oscillations. A_{OSC} of any vibrational modes can be obtained by fitting the residuals to a damped sine function. Relative A_{OSC} between different modes for comparison purposes, can be obtained from the relative FFT amplitudes.

The wavelength-resolved transient absorption dynamics of Ref-P and TS-P3 are shown in Supplementary Fig. 11. These data display clear oscillatory profiles, consistent with exciton–vibrational coupling. The maps also highlight distinct excited-state behaviours shaped by polymer architecture, with TS-P3 exhibiting modified spectral features on the ultrafast timescale that can be attributed to the presence of the through-space PDI units.

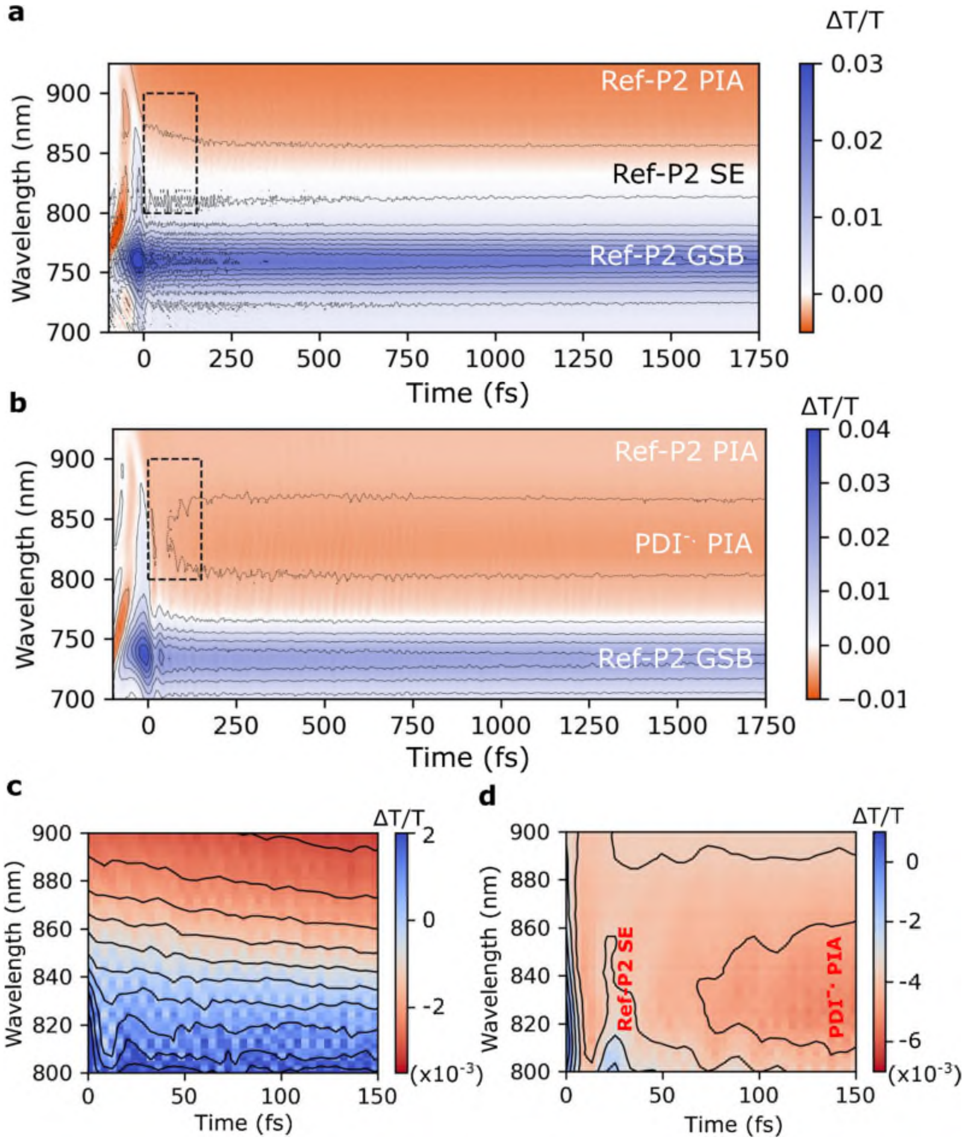

Supplementary Fig. 11 | Wavelength-resolved transient absorption dynamics of Ref-P and TS-P3 polymers: a, Transient absorption map of the Ref-P polymer, showing the temporal evolution of its photoexcited states. **b,** Transient absorption map of the TS-P3 space polymer, highlighting the influence of through-space PDI units on the excited-state dynamics. **c,d,** corresponding zoomed-in versions Ref-P(c) and TS-P3 (d).

Effective Huang–Rhys factor from band-integrated IVS: The high-frequency IVS contribution (1000–1500 cm^{-1}) was isolated by band-pass filtering the TA residuals after background

subtraction as shown in supplementary figure 12. An effective modulation depth m_{band} was defined as the RMS amplitude of the band-passed residual divided by the smooth TA background at the same probe wavelength. For Herzberg–Teller detection we used $S_{\text{eff}} \approx m_{\text{band}}$; for Franck–Condon detection we used $S_{\text{eff}} \approx m_{\text{band}}\bar{\Gamma}/(2\hbar\bar{\omega})$, where $\bar{\Gamma}$ is the representative half-width and $\bar{\omega}$ the band center. Measured amplitudes were corrected for the 12 fs Gaussian IRF using $H(\omega) = \exp[-\omega^2\sigma^2/2](\sigma = 5.1 \text{ fs})$. For a quick mode-resolved estimate, FFT peak areas within ±10 cm^{-1} of each mode were used to apportion S_{eff}into $S_i$via weights $w_i \propto$ FFT area, followed by mode-specific IRF corrections. Reported Svalues thus represent conservative (band-integrated) lower bounds on the high-frequency reorganization. From this analysis method we estimate effective S_i for high-frequency modes(1000-2000 cm^{-1}) is 0.52 and for the 1515 (±10) cm-1 mode the S_{eff} is 0.08 which is comparable to the previously reported BDT-DPP polymers[13,14].

To connect the vibronic coupling observed in the ultrafast photophysics, we carried out quantum dynamics simulations using the LVC Hamiltonian for the DPP–BDT and PDI fragments detailed in the Supplementary Note 21. This approach allows us to track in real time how nuclear motion along specific normal modes influences the evolution of the FE and CT states and the formation of the charge-separated configurations. By explicitly propagating the coupled electron–nuclear wavepacket, the simulations reveal the vibrational modes that most efficiently drive population transfer between these manifolds (Supplementary Fig.51). These simulations clearly show that several high-frequency modes of the DPP-BDT are strongly coupled with both the exciton as well as the cationic states (Supplementary Fig.50).

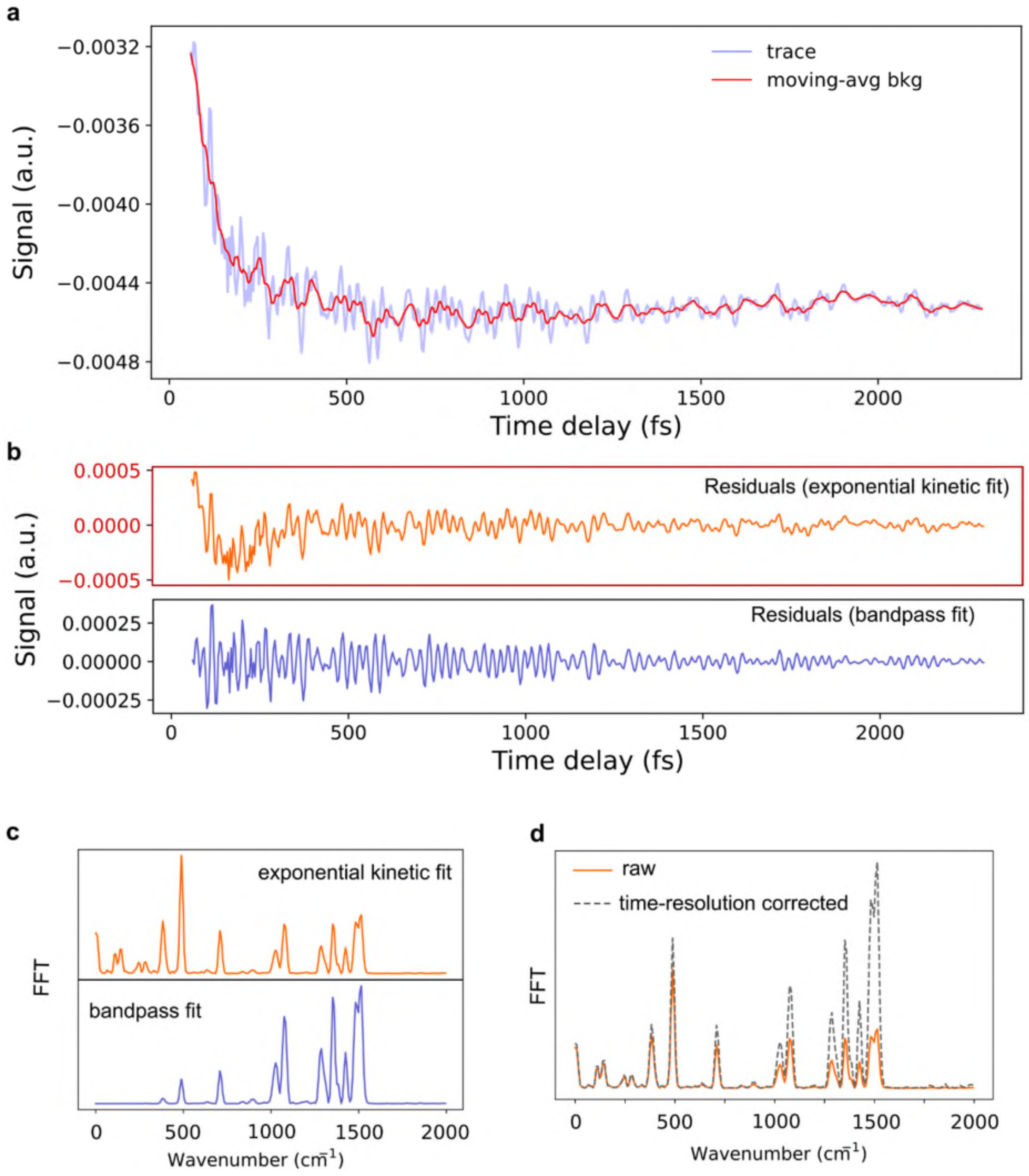

Supplementary Fig. 12 | Ultrafast kinetic traces, residuals, and corresponding Fourier transforms: a, Measured transient absorption kinetics (light blue) and the corresponding moving-average background (red, 200 fs window). **b,** Residuals obtained after subtracting the exponential kinetic fit (orange, top) and band-pass fit (blue, bottom), revealing coherent oscillations associated with vibrational modes. **c,** Fourier transforms (FFT) of the respective residuals, showing dominant vibrational modes in the 400–1600 cm^{-1} range. **d**, Comparison between the raw FFT spectrum (orange) and the time-resolution–corrected FFT (grey dashed), highlighting sharpening of vibrational peaks after correction.

Supplementary Note 8: picosecond transient absorption spectroscopy with the IR-probe (including the diluted space polymers)

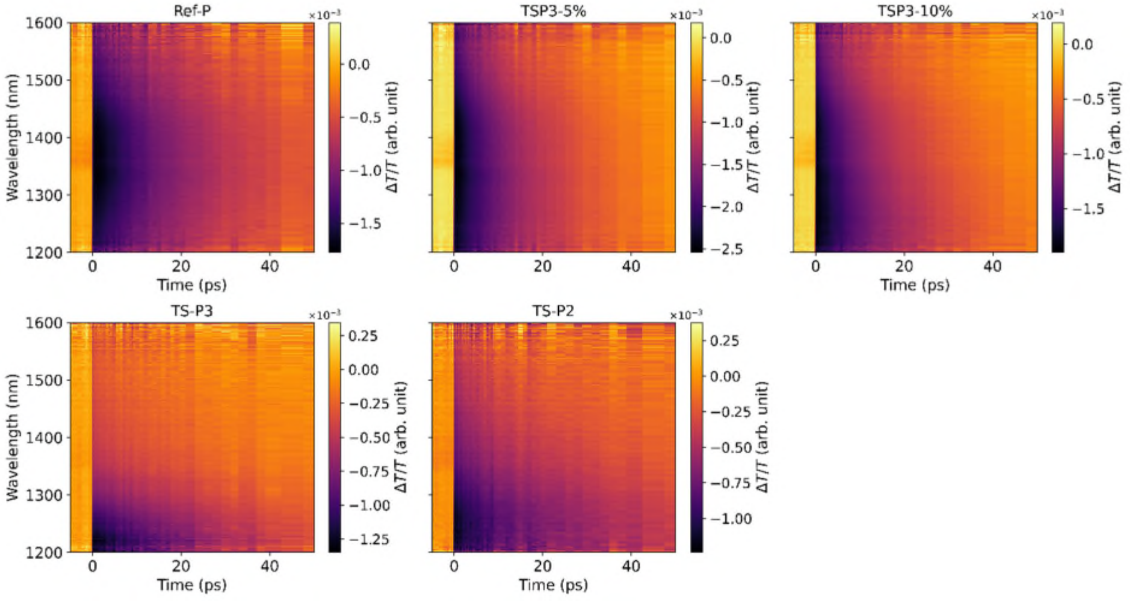

Supplementary Fig. 13 | Picosecond transient absorption spectroscopy with an IR probe: wavelength-resolved transient absorption kinetics measured using a 700 nm pump and an IR probe, capturing the excited-state dynamics on the picosecond timescale. Ref-P(top-left), TSP3-5%(top-middle), TSP3-10%(top-right), TS-P3 (bottom-left) and TS-P2 (bottom-right)

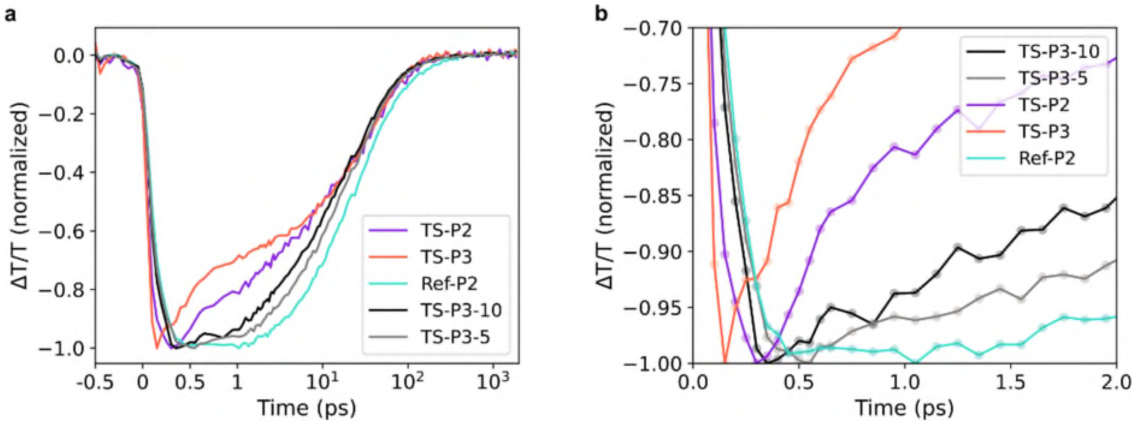

Supplementary Fig. 14 | Kinetic fitting of singlet exciton quenching in model heterojunctions: a, Sub-nanosecond transient absorption dynamics following 700 nm excitation of the polymer backbone chromophore, showing exciton quenching behavior across all model heterojunctions. **b**, Sub-2-picosecond dynamics highlighting ultrafast exciton quenching processes in both standard and diluted model heterojunctions.

As shown in the Supplementary Fig. 15, the singlet exciton quenching dynamics happen in sub-150 fs time scale which is the instrument limit of the IR-probed transient absorption spectroscopy used in this study supporting the sub vibrational time period electron transfer.

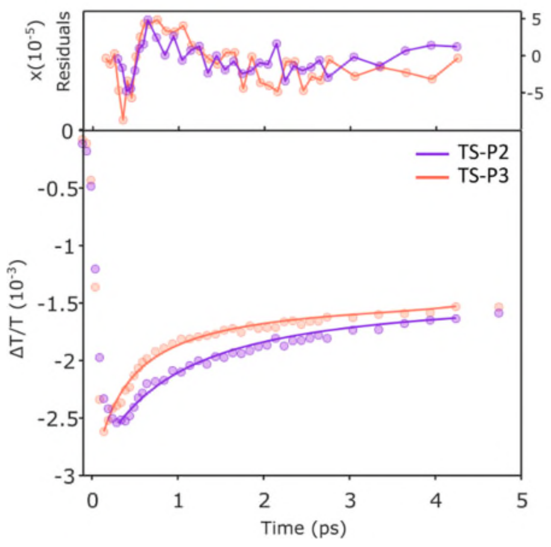

Supplementary Fig. 15 | Kinetic fitting of singlet exciton quenching in model heterojunctions, TS-P3 and TS-P2: Kinetic fitting of the singlet exciton quenching after photo-exciting the polymer backbone chromophore at 700 nm.

Supplementary Note 9: ~200 fs hole transfer dynamics from the PDI to the polymer backbone

As shown in Supplementary Fig. 16, a distinct hole polaron signature in the 1250–1300 nm region emerges upon excitation at both 700 nm (selectively exciting the polymer backbone) and 500 nm (preferentially exciting the PDI units). The appearance of this feature under 500 nm excitation may arise from one of several pathways: (i) energy transfer from the photoexcited PDI unit to the polymer backbone followed by electron transfer from the polymer to the PDI, (ii) direct hole transfer from the photoexcited PDI to the polymer, or (iii) weak excitation of a higher-energy band of the polymer backbone, leading to subsequent electron transfer to the PDI.

The temporal dynamics of the polymer's neutral singlet state (S_1), shown in Supplementary Fig. 17, reveal a slower quenching rate when excited at 500 nm, accompanied by a fast rise component. This suggests that ultrafast (sub-vibrational timescale) electron transfer is specific to the TS-P3 model heterojunction when the polymer backbone is directly excited. As shown in Supplementary Fig. 18a, a PDI radical anion–like feature appears upon 500 nm excitation of TS-P3 using an ultrafast pulse resonant with the PDI absorption. However, the formation of this anion is significantly slower compared to excitation at 700 nm, as evidenced by the kinetic trace in Supplementary Fig. 17d.

Moreover, Supplementary Fig. 18b shows a delayed rise (250–1000 fs) in the ground-state bleach of the polymer backbone upon PDI excitation—a feature absent when the polymer is directly excited (Supplementary Fig. 18c). This rise indicates a hole transfer from the photoexcited PDI to the polymer backbone. Kinetic analysis from Supplementary Figs. 14 and 15d supports this assignment, revealing that hole transfer from PDI to the DPP–BDT polymer backbone occurs on a ~200 fs timescale and is associated with a large energetic driving force (~800 meV). In contrast, the electron transfer from the photoexcited polymer to the PDI occurs in under 15 fs and is driven by a much smaller offset (<100 meV) between frontier orbitals.

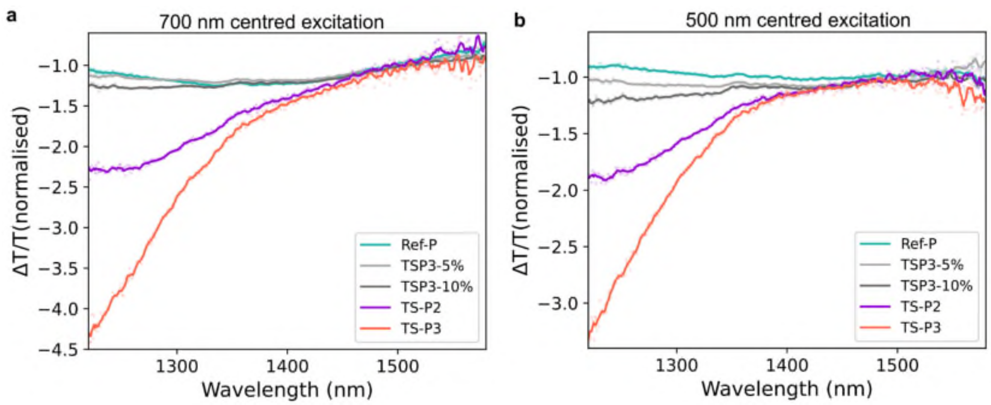

.

Supplementary Fig. 16 | IR probed TA spectra of the model heterojunction with variable photo-excitation IR- probed differential transient absorption spectra of the Ref-Polymer, TS-P2, and TS-P3 polymer ($CHCl_3$ solution) averaged over 0.5-1.5ps (normalized at 1500 nm) after photo-exciting polymer backbone chromophore with **a**, 700 nm pump (pulse width 200 fs), **b**, 500 nm pump (pulse width 200 fs)

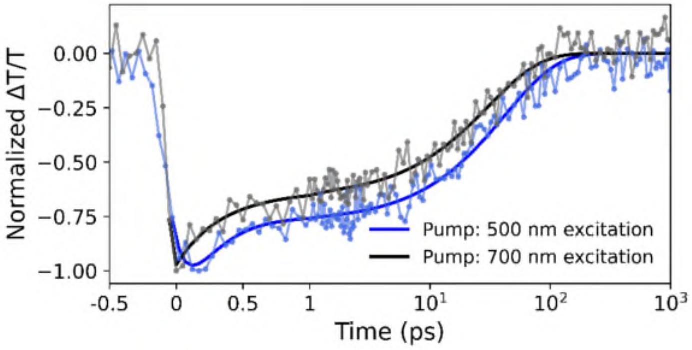

Supplementary Fig. 17 | Comparison of the polymer backbone's S1 state upon pumping with variable excitation in TS-P3 The kinetics are extracted from the probe wavelength regime 1500-1550 nm (corresponding to polymer backbone's S1→Sn transition)

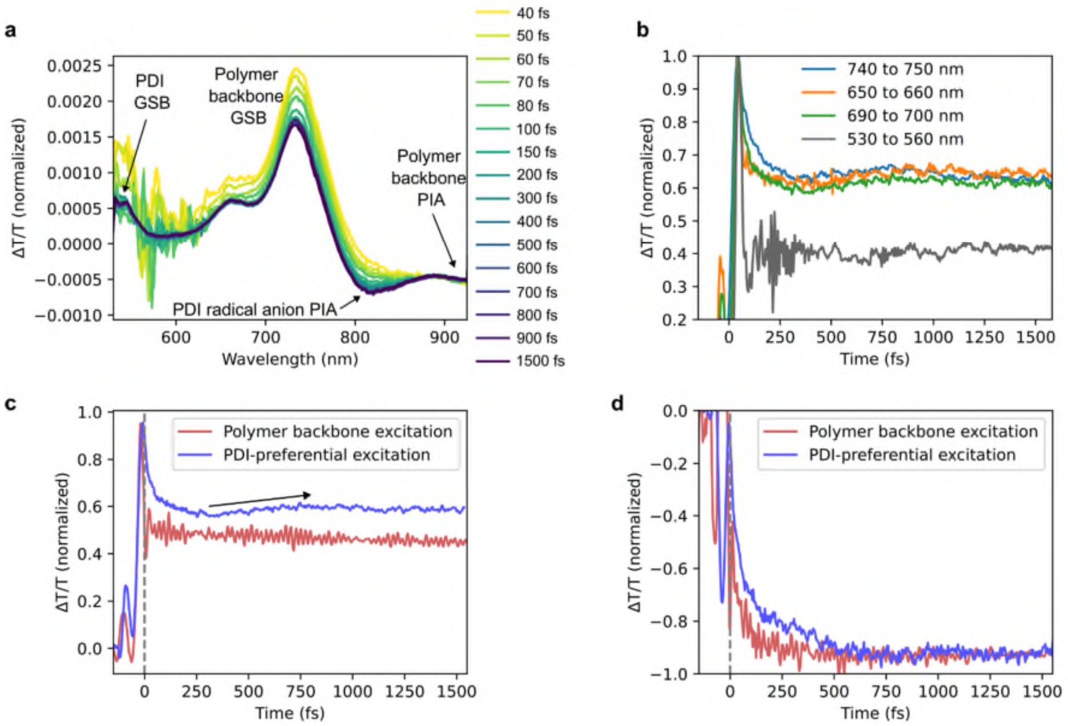

Supplementary Fig. 18 | Ultrafast transient Absorption spectroscopy with PDI-preferential excitation (550 nm centred) : a, differential transmission spectra at different time delays Ultrafast transient Absorption spectroscopy on TS-P3 with PDI-preferential excitation (550 nm centred): a, differential transmission spectra (ΔT/T) at different time delays, b, The temporal kinetics extracted from different probe wavelength regime: polymer backbone chromophore GSB regime (650-660, 690-700, 740-750 nm) and PDI GSB regime (530-560 nm). c, Kinetics extracted from the polymer backbone chromophore GSB regime (690-700 nm) after specifically exiting the polymer backbone (red pump, red curve) and preferentially exciting the PDI (green pump, blue curve). d, Kinetics extracted from the probe wavelength regime 800-810 nm (PDI radical anion absorption regime) after specifically exiting the polymer backbone (red pump, red curve) and preferentially exciting the PDI (green pump, blue curve).

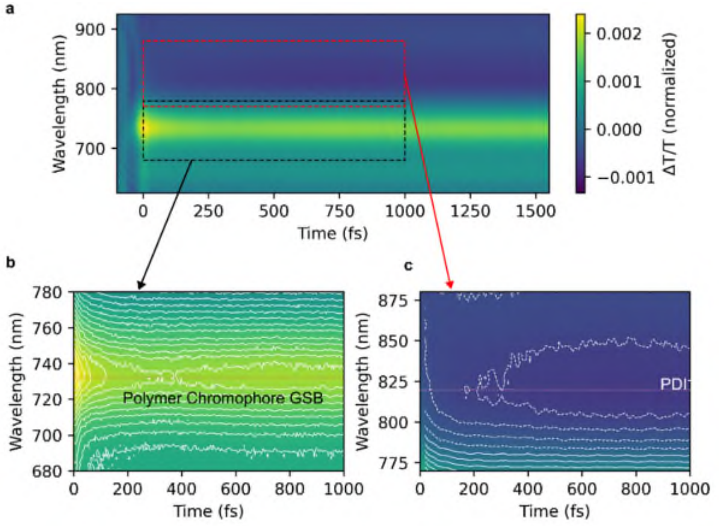

Supplementary Fig. 19 | Wavelength resolved transient absorption map of TS-P3 with PDI-preferential excitation (550 nm centred): Dynamics of PDI radical anion formation is highlighted in the panel c. Panels a–c share the same colour scale, shown in panel a.

Supplementary Note 10: (Pre)Resonant Impulsive vibrational spectroscopy of the PDI with an resonant probe

To directly probe the vibrational coherence associated with the PDI chromophore, we performed resonant impulsive vibrational spectroscopy using a sub-12 fs broadband pulse centered at 550 nm, partially overlapping with the PDI absorption. As shown in Supplementary Fig. 20a, the excitation profile was partially matched to the absorption spectrum of PDI-OMe in chloroform. The resulting transient absorption dynamics (Supplementary Fig. 20b) reveal prominent oscillatory modulations superimposed on the excited-state response, indicative of coherent vibrational wavepacket motion. Fourier-transform analysis (Supplementary Fig. 20c) highlights well-defined frequency components across the probe spectrum, with a mode at ~1293 cm^{-1} along with 1380, 1555 and 1590 cm^{-1}. Those modes, corresponding to a characteristic PDI skeletal vibration, are clearly resolved in the integrated Fourier spectrum (Supplementary Fig. 20d).

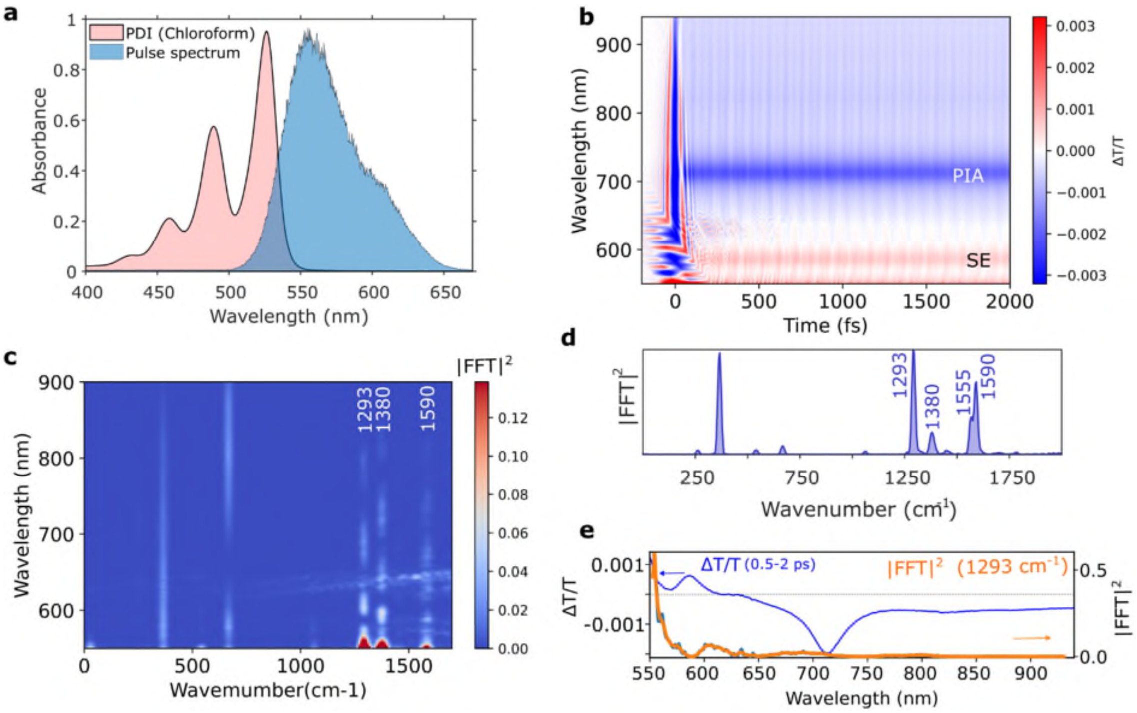

Supplementary Fig. 20 | Resonant impulsive vibrational spectroscopy of PDI ($ChCl_3$) : a, Absorption spectra of the PDI-OMe and spectrum of the impulsive pulse for resonant excitation. **b,** Contour plot of the transient absorption measured on PDI by photoexciting with the 550nm centred broadband pulse presented in (subfig. a). **c,** Probe wavelength resolved Fourier transformed maps. **d,** Integrated Fourier transformed spectra for the wavelength range λ = 665-685 nm. **e,** $|FFT|^2$ amplitude (1293 cm^{-1}) as a function of the wavelength (orange, right Y axis) and ΔT/T averaged over 500-2000 fs (blue line, left Y axis)

Supplementary Note 11: Resonant Impulsive vibrational spectroscopy of the chemically reduced PDI radical anion with an resonant probe

To investigate the vibrational dynamics of the charge-separated state, we performed resonant impulsive vibrational spectroscopy on the chemically reduced PDI-OMe radical anion in chloroform. As shown in Supplementary Fig. 21a, the broadband excitation pulse (centered at 700 nm) was tuned to overlap with the distinct absorption features of the radical anion. The resulting transient absorption data (Supplementary Fig. 21b) exhibit coherent oscillatory features superimposed on the excited-state signal, indicating the generation of vibrational wavepackets.

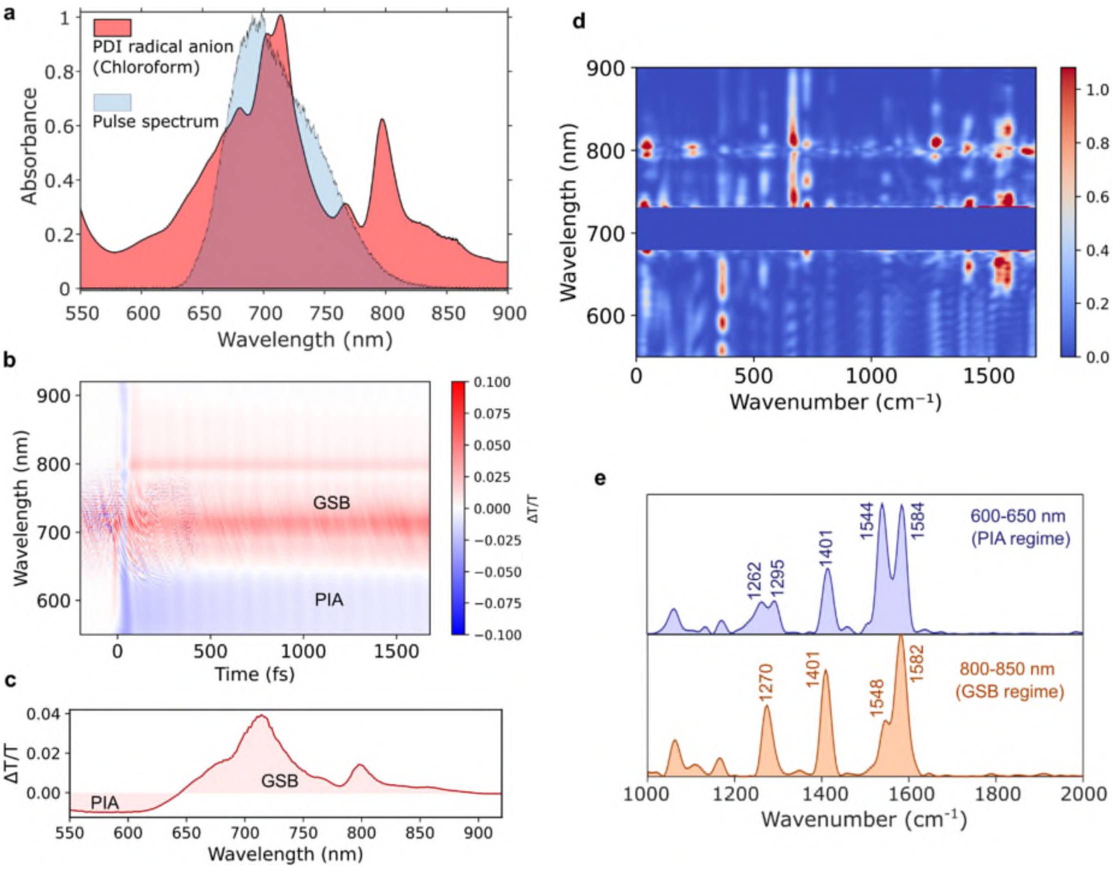

Supplementary Fig. 21 | Resonant impulsive vibrational spectroscopy directly on the chemically reduced PDI radical anion ($ChCl_3$) : a, Absorption spectra of the PDI-Ome radical anion and spectrum of the impulsive pulse for resonant excitation. **b,** Contour plot of the transient absorption measured on PDI-Ome radical anion by photoexciting with the 700nm centred broadband pulse presented in (subfig. a). **c,** ΔT/T averaged over 500-2000 fs. **d,** Probe wavelength resolved Fourier transformed maps. **e,** Integrated Fourier transformed spectra for the wavelength range λ = 600-650 nm (PIA regime, blue) and 800-850 nm (GSB regime, orange).

Time-averaged differential transmission (ΔT/T) in the 500–2000 fs window (Supplementary Fig. 21c) highlights persistent photoinduced absorption and ground-state bleach signals. Fourier transform analysis (Supplementary Figs. 21d,e) reveals distinct vibrational modes in both

the PIA (600–650 nm, blue) and GSB (800–850 nm, orange) regions, suggesting that vibrational coherence persists in both excited and ground electronic configurations of the radical anion. These measurements establish the mode-specific vibrational landscape of the PDI radical anion, providing a reference point for interpreting coherent dynamics observed in the directly impulsively excited PDI radical anion state.

Supplementary Note 12: Off-resonant Impulsive vibrational spectroscopy of the PDI

In the Supplementary Figure 22a, we observe the absorption spectra of the PDI molecule in chloroform, where the onset is at 556 nm. The 700 nm-pulse (depicted in red) is electronically off-resonant with respect to PDI. Notably, the broadband 700 nm pulse is also off-resonant with the solvent, chloroform, which has an absorption onset at 250 nm[15]. Consequently, the broadband pulse is capable of inducing vibrational coherence in the ground electronic state of both PDI and chloroform (off-resonant vibrational coherence).

Intriguingly, as illustrated in Supplementary Figure 22b, the vibrational modes of chloroform (263, 366, 669 cm^{-1}) exhibit a distinctly different intensity pattern with respect to the vibrational modes of PDI (1060, 1295, 1374, 1455, 1586 cm^{-1}), depending on the probe wavelength. The off-resonant vibrational coherence (VC) of chloroform does not exhibit drastic variations in mode intensities across different probe wavelengths. Conversely, the VC of PDI displays a predominant intensity in the bluer regime (550-600 nm), where the probe resonates with the $S_0 \rightarrow S_1$. It is important to note that there might be a lowering in the effective energy gap between the excited and ground states when a superposition of vibrational eigenstates is generated in the ground electronic state of PDI.

This phenomenon can be explained by the enhancement of the detection of the wavepacket generated in the electronic ground state due to resonant probing. When the probe is off-resonant, the coherent synchronous molecular motions of the system understudy induces a time dependent periodic change in the macroscopic polarization which in turn correlated to the macroscopic property, refractive index (η). As shown in Supplementary Fig. 22c, η shows a periodic change with time where τ is the time period of the wavepacket motion. Now interaction of the non-resonant optical probe pulse can lead blue shift and red shift the probe energy for the negative and positive slope of the refractive index (η) with time respectively.

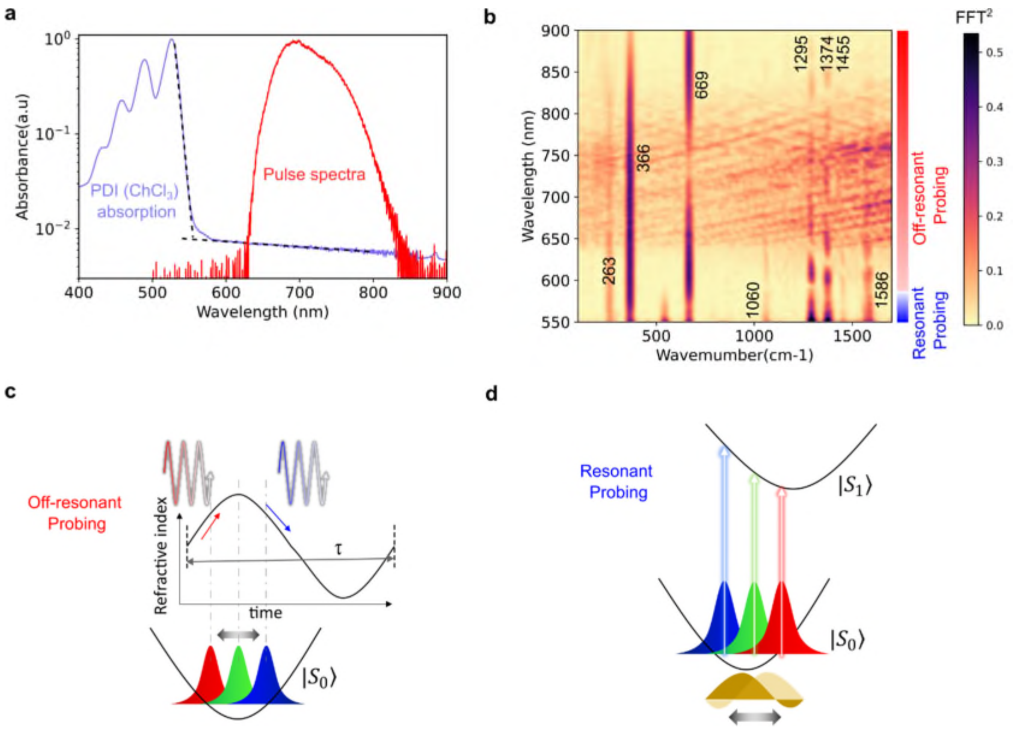

Supplementary Fig. 22 | Off-resonant impulsive vibrational spectroscopy of PDI ($ChCl_3$) : a, Absorption spectra of the neutral PDI (blue) and spectral profile of the off-resonant pulse (red). **b,** Probe wavelength resolved IVS spectra of the off-resonant wavepacket generated on the ground state. **c,d,** Mechanism of the detection of the coherent oscillation generated on the ground electronic state by the **c,** off-resonant Probe and **d,** resonant probe.

When $\frac{d\eta}{dt}$ is positive, the phase velocity of later part of the probe will be slower, shifting the probe energy to the red in frequency domain. Similarly, when $\frac{d\eta}{dt}$ is negative, the phase velocity of later part of the probe will be faster shifting the probe energy to the blue in frequency domain (Supplementary Fig. 22c). Thus, time domain modulation of macroscopic refractive index induced by coherent wavepacket motion, can modulate the probe energy in the frequency domain. Detection of oscillatory signature with a resonant probe is easier to visualize (Supplementary Fig. 22d). The created coherent nuclear wavepacket moves back and forth along the vibrational coordinate changing the energy gap for the vertical transition in oscillatory manner which subsequently redshift and blueshift the probe absorption accordingly. Thus, impulsively generated time domain VC oscillations can be imprinted in the spectral domain of a time delayed probe. The encoded vibration signal can be resolved by a spectrally resolved spectrometer. Thus, the enhanced detectability of the wavepacket generated in the ground state through resonant probing leads to a distinctive observation: off-resonant modes of the PDI generated in the S_0 state exhibit higher amplitudes in the bluer region of the probe

spectrum, which resonates with the electronic absorption. This distinct pattern is consistently observed for the vibrational modes at 1060, 1295, 1374, 1455, and 1586 cm^{-1}.

To investigate the vibrational coherence pathways associated with ground-state PDI units in a polymer–PDI environment, we performed resonant impulsive vibrational spectroscopy on a physical mixture of Ref-P and PDI, with the PDI content matched to that in TS-P3. Upon excitation with a broadband pulse centered at 700 nm, the transient absorption map (Supplementary Fig. 23a) and corresponding wavelength-resolved spectra (Supplementary Fig. 23b) exhibit dynamics comparable to those observed in Ref-P. The kinetic traces extracted from the 790–830 nm region (Supplementary Fig. 23c) primarily reflect the ground-state bleach of the polymer backbone. However, the probe-wavelength-resolved impulsive vibrational spectra (Supplementary Fig. 23d) show no evidence of the 1283 cm^{-1} mode that is characteristic of TS-P3.

The absence of this mode in the physical mixture indicates that no diffusion-mediated electron transfer occurs from the polymer to the PDI units under these conditions. Furthermore, the data suggest that there is no pre-formed ground-state complex between the polymer and PDI that can be optically accessed by the red impulsive excitation. The lack of the 1293 cm^{-1} vibrational mode—distinctly observed in TS-P3—also confirms that it is not a result of off-resonant excitation of an isolated PDI unit. Taken together, these findings demonstrate that the vibrational coherence observed in TS-P3 is a consequence of ultrafast sub-vibrational time period electron transfer, which does not exist in the physical mixture.

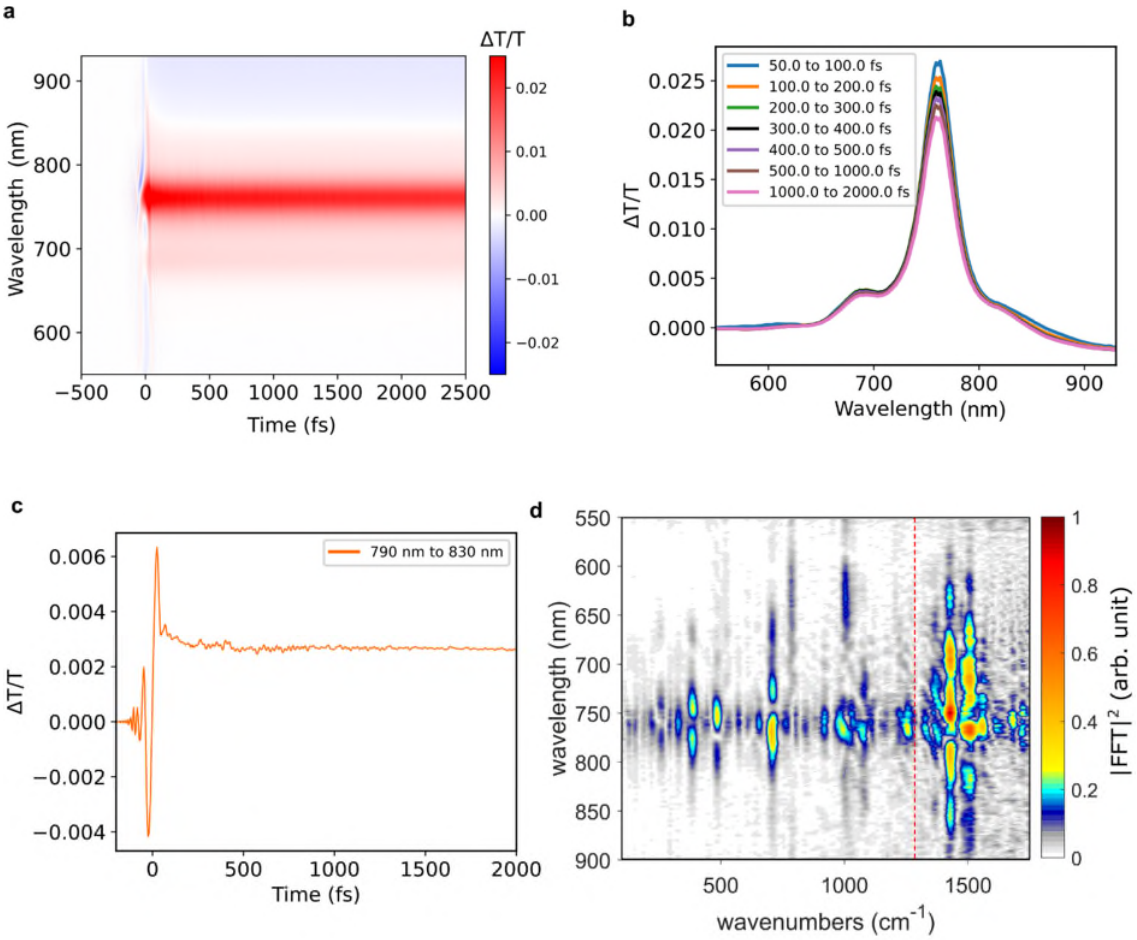

Supplementary Fig. 23 | Resonant impulsive vibrational spectroscopy on the chemical mixture of the Ref-P and PDI maintaining the same number of PDI content per BDT-DPP unit as TS-P3 (excitation by the 700 nm centred broadband pulse) : a, Transient absorption (ΔT/T) map. b, Transient absorption spectra (ΔT/T) plotted against probe wavelength (nm) for different pump-probe delay. **c,** Transient absorption kinetics extracted from the 790-830 nm. d, Probe wavelength resolved IVS map. The red dotted line highlights the position of the 1283 cm^{-1}.

Supplementary Note 13: Additional Evidence Against Direct Excitation of PDI by Red-Shifted Impulsive Pump

To further understand the role of polymer–PDI coupling in modulating vibrational coherence, we compared the PDI GSB vibrational signals between Ref-P and TS-P3 following resonant excitation of the polymer backbone (Supplementary Fig. 24). As shown in Supplementary Fig. 24a–b, the raw ΔT/T traces (top panels) reveal coherent modulations superimposed on the electronic response. After deconvolution of the population dynamics, the residual vibrational coherence (middle panels) displays significantly enhanced amplitudes. The corresponding FFT power spectra (bottom panels) reveal similar vibrational mode profiles in both Ref-P and TS-P3 within the PDI ground-state bleach (GSB) regime. This indicates that the observed modes

originate from the polymer backbone chromophore rather than from the PDI units. The absence of distinct PDI vibrational modes in this spectral region further supports the conclusion that the charge-transfer (CT) complex is not directly excited. Consequently, the vibrational coherence observed at 1283 cm^{-1} (main) in TS-P3 does not arise from direct excitation of a CT state, but rather reflects electron transfer-derived mode, selectively coupled in the electronically interacting heterojunction environment.

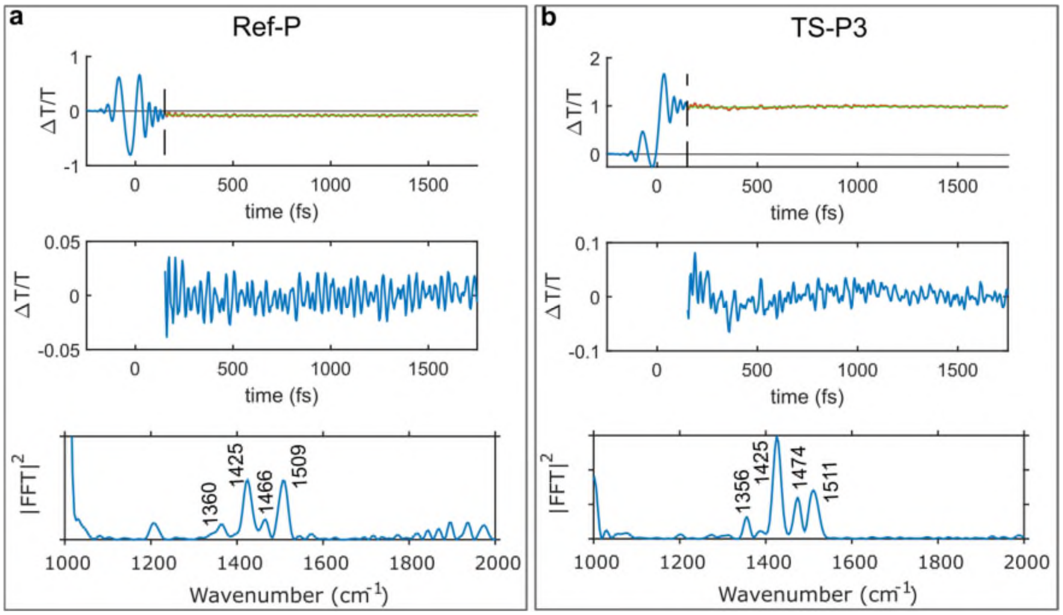

Supplementary Fig. 24 | Vibrational coherence extracted from the PDI ground-state bleach regime in TS-P3 in comparison to the Ref-P following impulsive excitation of polymer backbone chromophore: Data plotted for a Ref-P, b TS-P3. (top) Normalised ΔT/T extracted from the PDI GSB, (middle) Corresponding vibrational coherence after deconvoluting the electronic dynamics, (bottom) FFT power spectrum from the obtained vibrational coherence

Supplementary Note 14: Description of the anharmonicity in the impulsive excitation

As shown in Supplementary Fig. 25a, the vibrational ground state is represented as $\psi_\alpha(r)$ and the first and second excited vibrational states are $\psi_\beta(r)$ and $\psi_\gamma(r)$ respectively, in the electronic excited state manifold (S_1). After impulsive photoexcitation by broadband laser pulse from the electronic ground state (S_0), if the simultaneous occupation happens for all three vibrational states, then the newly generated non-stationary states can be represented as-

$$\psi(r,t) = c_\alpha(t)e^{-i\omega_\alpha t}\psi_\alpha(r) + c_\beta(t)e^{-i\omega_\beta t}\psi_\beta(r) + c_\gamma(t)e^{-i\omega_\gamma t}\psi_\gamma(r) \qquad \text{(S8)}$$

where c_α, c_β, c_γ corresponds to the contribution of the each vibrational state to the non-eigen state. Hence, for a narrow-band, non-impulsive excitation (no coherent superposition of states) then $c_\alpha = 0$, c_β, $c_\gamma = 0$. It is noteworthy that the $\psi(r,t)$ has an exponential damping term as well which stands for the dephasing.

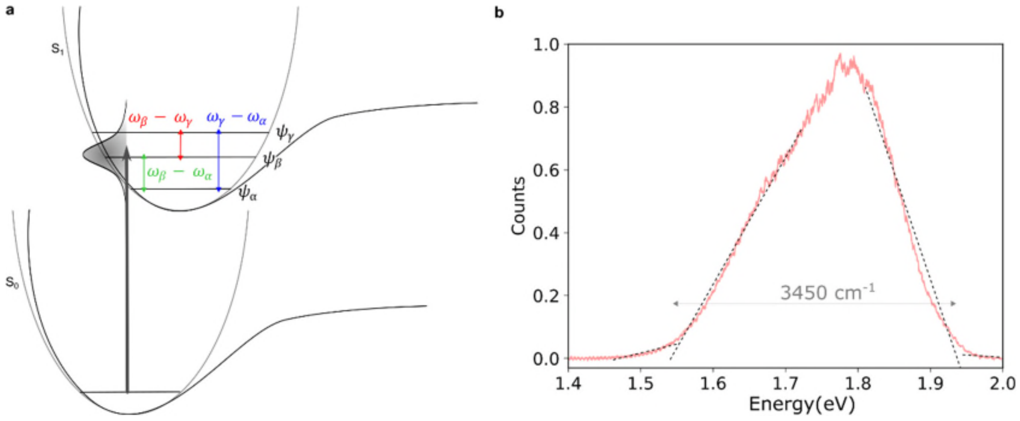

Supplementary Fig. 25 | Effect of the anharmonicity in the impulsive excitation : a, Superposition of the vibrational states involving multiple quantum numbers. b, The spectral profile of the pump

The time-dependent molecular polarization can be described as -

$$P(t) = \langle \psi(r,t)|\mu|\psi(r,t)\rangle \text{(S9)}$$

where μ is the dipole moment operator.

$$P(t) = \langle c_\alpha e^{-i\omega_\alpha t}\psi_\alpha(r) + c_\beta e^{-i\omega_\beta t}\psi_\beta(r) + c_\gamma e^{-i\omega_\gamma t}\psi_\gamma(r)|\mu|c_\alpha e^{-i\omega_\alpha t}\psi_\alpha(r) + c_\beta e^{-i\omega_\beta t}\psi_\beta(r) + c_\gamma e^{-i\omega_\gamma t}\psi_\gamma(r)\rangle \quad \text{(S10)}$$

$$P(t) = c_\alpha^* c_\beta \mu_{\alpha\beta} e^{-i(\omega_\beta - \omega_\alpha)t} + c_\beta^* c_\alpha \mu_{\beta\alpha} e^{i(\omega_\beta - \omega_\alpha)t} + c_\gamma^* c_\beta \mu_{\gamma\beta} e^{-i(\omega_\beta - \omega_\gamma)t} + c_\beta^* c_\gamma \mu_{\beta\gamma} e^{i(\omega_\beta - \omega_\gamma)t} + c_\alpha^* c_\gamma \mu_{\alpha\gamma} e^{-i(\omega_\gamma - \omega_\alpha)t} + c_\gamma^* c_\alpha \mu_{\gamma\alpha} e^{i(\omega_\gamma - \omega_\alpha)t} \text{(S11)}$$

$$P(t) = \mu_{\alpha\beta}(c_\alpha^* c_\beta e^{-i(\omega_\beta - \omega_\alpha)t} + c_\alpha c_\beta^* e^{i(\omega_\beta - \omega_\alpha)t}) + \mu_{\gamma\beta}(c_\gamma^* c_\beta e^{-i(\omega_\beta - \omega_\gamma)t} + c_\gamma c_\beta^* e^{i(\omega_\beta - \omega_\gamma)t}) + \mu_{\alpha\gamma}(c_\alpha^* c_\gamma e^{-i(\omega_\gamma - \omega_\alpha)t} + c_\alpha c_\gamma^* e^{i(\omega_\gamma - \omega_\alpha)t}) \quad \text{(S12)}$$

The macroscopic polarization can be represented as $P_N(t) = N.P(t)$, where N is the number of molecules. Hence, the vibrational coherence generated by the superposition of three vibrational states can oscillate with three frequencies $(\omega_\beta - \omega_\alpha)$, $(\omega_\beta - \omega_\gamma)$ and $(\omega_\gamma - \omega_\alpha)$.

$$|\omega_\beta - \omega_\alpha| = \left\{\omega_e\left(1+\tfrac{1}{2}\right) - \chi_e\omega_e\left(1+\tfrac{1}{2}\right)^2\right\} - \left\{\omega_e\left(0+\tfrac{1}{2}\right) - \chi_e\omega_e\left(0+\tfrac{1}{2}\right)^2\right\} = \omega_e - 2\chi_e\omega_e \quad \text{(S13)}$$

$$|\omega_\gamma - \omega_\beta| = \left\{\omega_e\left(2+\frac{1}{2}\right) - \chi_e\omega_e\left(2+\frac{1}{2}\right)^2\right\} - \left\{\omega_e\left(1+\frac{1}{2}\right) - \chi_e\omega_e\left(1+\frac{1}{2}\right)^2\right\} = \omega_e - 4\chi_e\omega_e \qquad \text{(S14)}$$

For a pure harmonic potential surface ($\chi_e = 0$), $|\omega_\beta - \omega_\alpha| = |\omega_\beta - \omega_\gamma|$ and the harmonic frequency, $|\omega_\gamma - \omega_\alpha| = 2.|\omega_\beta - \omega_\alpha|$

But for anharmonic potential, $|\omega_\beta - \omega_\alpha| - 2\chi_e\omega_e = |\omega_\beta - \omega_\gamma|$.

As a result of that, impulsive population to multiple (> 2) vibrational states in an anharmonic potential surface can lead to redshift of the fundamental frequency as experimentally observed before for formamidinium based lead halide perovskite nanocrystals (PNCs)[16].

As described above impulsive superposition involving >2 vibrational states should give rise observation of wavepacket having higher harmonic frequencies and that can be experimentally observed for the low-frequency modes. As shown in the Supplementary Fig. 25 b, the spectral coverage of the broadband excitation source used for the impulsive excitation of the polymer backbone used is 3450 cm^{-1}.

In our experiment along with loss in the vibrational coherence, we can additionally observe a noticeable 12 cm^{-1} blue shift of the 1507 cm^{-1} mode in TS-P3 space-polymer (Figure 3k, main). This frequency shift is not an outcome of the time-domain phase interference with neighbouring modes as investigated in the later part of this supplementary note. It is noteworthy that the resonance cw-Raman reveals similar frequencies for this mode for all there space polymers (Figure 3l, main).

Considering the broadband spectral coverage of the impulsive pump (3450 cm^{-1}, see Supplementary Information n-viii), superposition of the 3 vibrational quantum states (along 1512 cm^{-1} coordinate) can lead to generation of the non-eigen state, $\psi(r,t)$.

$$\psi(r,t) = c_0(t)e^{-i\omega_0 t}\psi_0(r) + c_1(t)e^{-i\omega_1 t}\psi_1(r) + c_2(t)e^{-i\omega_2 t}\psi_2(r)$$

Where $\psi_0(r)$, $\psi_1(r)$ and $\psi_2(r)$ are the vibrational eigen states with respective frequencies ω_0, ω_1 and ω_2 and $c_0(t)$, $c_1(t)$ and $c_2(t)$ are the corresponding weightage of those states to the non-stationary state produced by the 'impulsive red pump'.

Hence the time-dependent macroscopic polarization (with N molecule) induced by this wavepacket can be represented as $NP(t)$

$$P(t) = \langle\psi(r,t)|\hat{\mu}|\psi(r,t)\rangle$$

$= \mu_{\alpha\beta}(c_\alpha^* c_\beta e^{-i(\omega_\beta-\omega_\alpha)t} + c_\alpha c_\beta^* e^{i(\omega_\beta-\omega_\alpha)t}) + \mu_{\gamma\beta}(c_\gamma^* c_\beta e^{-i(\omega_\beta-\omega_\gamma)t} + c_\gamma c_\beta^* e^{i(\omega_\beta-\omega_\gamma)t}) +$
$\mu_{\alpha\gamma}(c_\alpha^* c_\gamma e^{-i(\omega_\gamma-\omega_\alpha)t} + c_\alpha c_\gamma^* e^{i(\omega_\gamma-\omega_\alpha)t}) \quad (S15)$

For a pure harmonic potential surface, $|\omega_\beta - \omega_\alpha| = |\omega_\beta - \omega_\gamma|$ and for anharmonic potential, $|\omega_\beta - \omega_\alpha| - c = |\omega_\beta - \omega_\gamma|$, c is the anharmonicity term. As a result of that impulsive population to the multiple vibrational state (n≥3) in an anharmonic potential surface can give rise to redshifted vibrational frequencies which experimentally observed before for perovskite nanocrystals[17].

Now vectorial displacement along the driving vibrational mode prepare the transition state for the electron transfer. And different vibronic levels have different pathways with different energy off-set and of electron transfer which can be picturised by the 'nesting'[18,19] of the potential energy surfaces. As a result of that $\{c_\alpha(t), c_\beta(t), c_\gamma(t)\}$ rearranges their value and dispersion of the initially photogenerated wavepacket happens which explains the drop in the coherence for the driving mode in TS-P3. We hypothesize the blue shift in the frequency of the driving mode is due to the participation of the 'hot' vibrational states (maximum decrease in the $c_\gamma(t)$ parameter) and lowering the weightage of the anharmonic levels in the overall wavepacket.

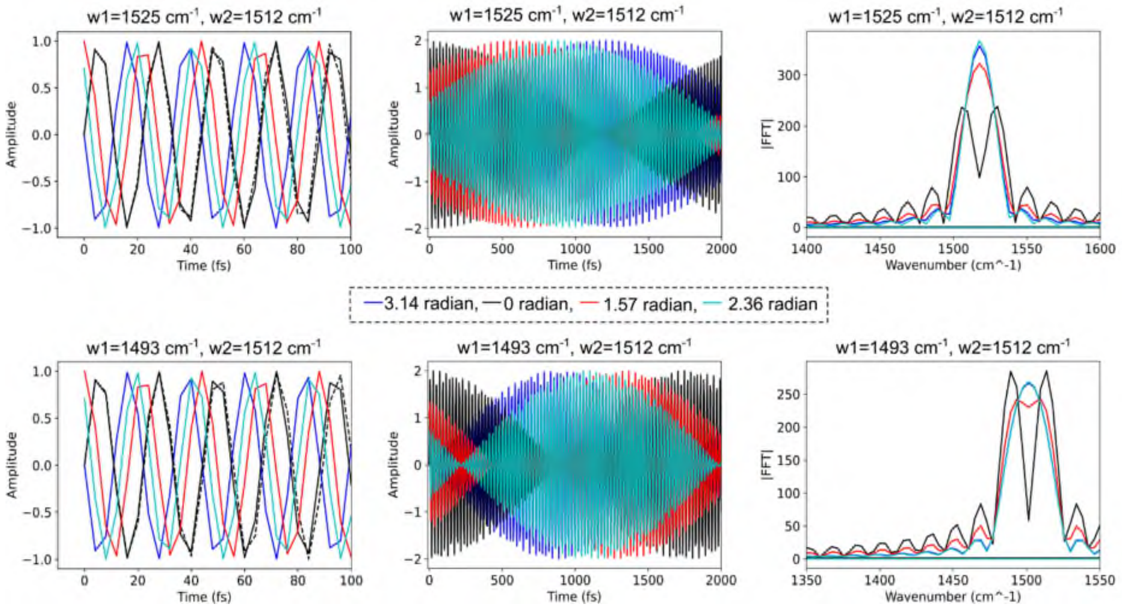

Supplementary Fig. 26 | Fourier transformation artifact from relative phase difference of the two vibrational mode with close frequency < 20 cm−1): Left column figures show the mode with w2 frequency (black dotted line) with zero phase and four solid lines representing the mode with w1 frequency at four different phase difference (0, 1.57, 2.36, 3.14 radian). The figures in the middle column show the summed oscillation of w1 (phase = 0) and w2(ϕ) for different values of ϕ (ϕ = 0, 1.57, 2.36, 3.14).The right column figures show the FFT spectra of the summed oscillation with different phase differences. This is a raw FFT with no windowing or zero-padding, and the data points are spaced at 4fs to match the experimental conditions

To illustrate potential frequency shifts arising from Fourier transform (FFT) artifacts, we simulated the FFT of two sine wave oscillations with closely spaced, arbitrary frequencies, leading to partial spectral overlap (Supplementary Fig. 26). By varying the relative phase between the two signals from 0 to π, we observed shifts in the apparent peak positions within the frequency bands. This exercise highlights a key limitation: extracting precise oscillation frequencies and relative intensities from the absolute FFT amplitude can be error-prone. Specifically, (i) the FFT peak positions may not accurately reflect the true oscillation frequencies, and (ii) the spectral line shape depends on the phase relationship between components, making it difficult to fit the spectrum using standard models such as a sum of Lorentzian functions.

To evaluate whether this artifact affects our experimental results, we applied the same analysis to experimentally observed vibrational signals (Supplementary Fig. 27). In this case, the frequency components were more well-separated, and the artifact was significantly reduced. Furthermore, we assessed the impact of varying the FFT window start time (Supplementary Fig.28), and found no appreciable shift in frequency. These findings confirm that the ~12 cm^{-1} redshift observed for the 1507 cm^{-1} mode in TS-P3 is not an artifact of the Fourier transform analysis.

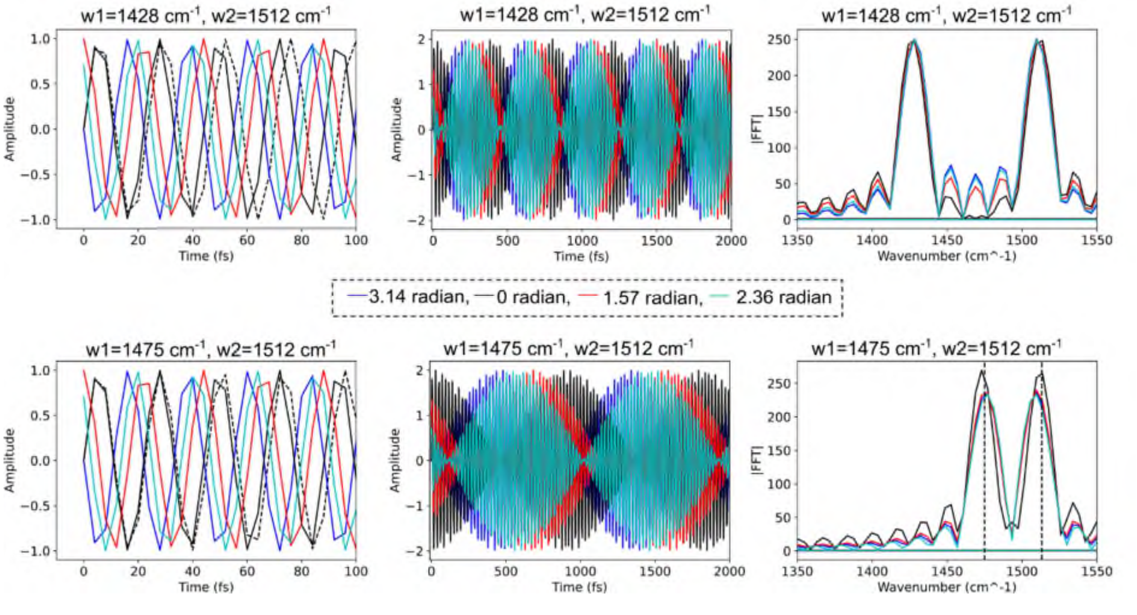

Supplementary Fig. 27 | Fourier transformation artifact from the relative phase difference of the two experimentally obtained vibrational modes with neighbouring frequencies: Left column figures show the mode with w2 frequency (black dotted line) with zero phase and four solid lines representing the mode with w1 frequency at four different phase difference (0, 1.57, 2.36, 3.14 radian). The figures in the middle column show the summed oscillation of w1 (phase = 0) and w2(ϕ) for different values of ϕ (ϕ = 0, 1.57, 2.36, 3.14).The right column figures show the FFT spectra of the summed of oscillation

with different phase differences. This is a raw FFT with no windowing or zero padding, and the data points are spaced at 4fs to match the experimental conditions.

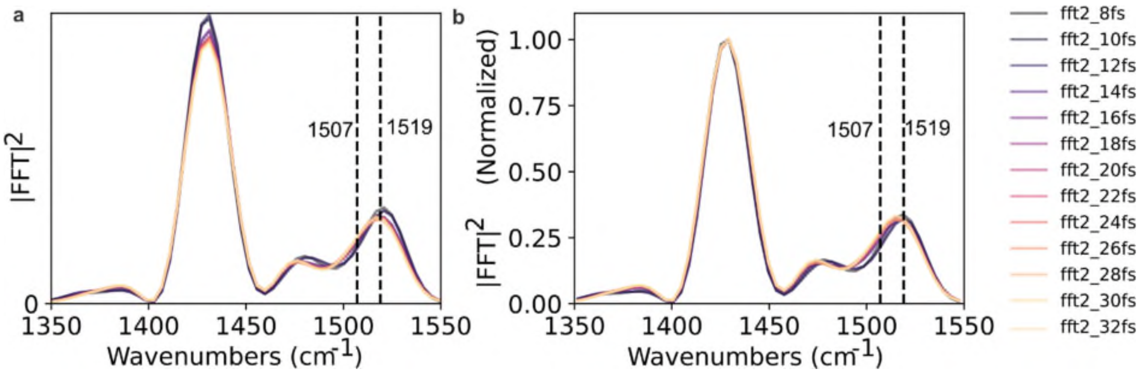

Supplementary Fig. 28 | Sliding window Fourier transformation of the experimentally obtained vibrational coherence of TS-P3: a, Fourier power spectra, b, normalized Fourier power spectra. The window length is 1.5 ps with the starting data-point at 8, 10, 12, 14, 16, 18, 20, 22, 24, 26, 28, 30, 32 fs. Two vertical dotted lines correspond to frequencies 1507 and 1519 cm^{-1} for reference.

Supplementary Note 15: Quantum chemical calculations

The ground state structure of a representative molecular TS-P3 dimer was optimized at the Density Functional Theory (DFT) level with the ωB97X-D exchange-correlation functional and the 6-31G(d,p) basis set. Alkyl chains were removed to reduce the computational cost. The optimized structure was then subjected to a frequency analysis, along with a Raman spectrum calculation with the same level of theory, where frequencies were scaled by a factor of 0.949 to account for anharmonicity. The most intense Raman modes in the 1400-1550 cm^{-1} region were analysed by quantifying the relative weight of the displacement over the different molecular units of the TS-P3 dimer: BDT, DPP, and PDI.

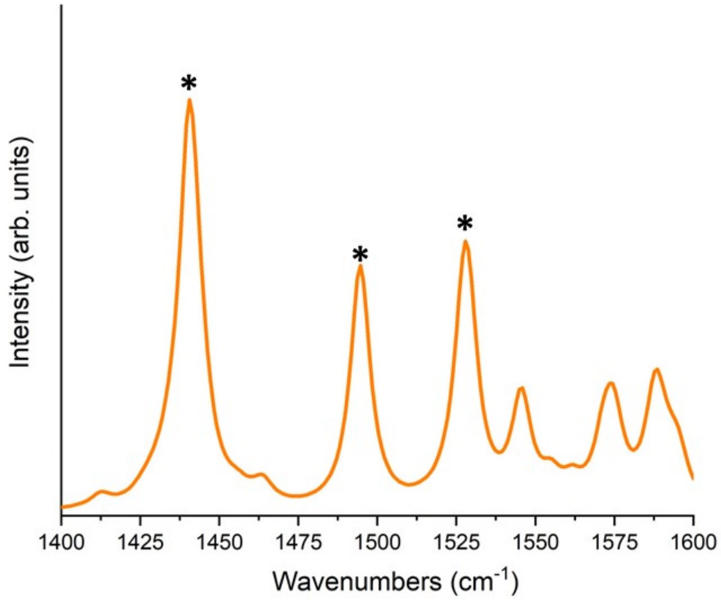

Supplementary Fig. 29 | Raman spectrum of the molecular TS-P3 dimer: Asterisks mark the vibrational modes at 1440, 1495, and 1527 cm⁻¹, which closely correspond to the experimentally observed bands at 1429, 1482, and 1512 cm⁻¹, respectively.

Supplementary Table S2 | Relative vibrational displacement contributions across molecular subunits of the TS-P3 dimer: Tabulated weights of atomic displacements associated with each molecular unit. "L" and "R" denote the left and right monomers of the TS-P3 dimer, respectively.

	**1440 cm^{-1}**	**1441 cm^{-1}**	**1444 cm^{-1}**	**1495 cm^{-1}**	**1527 cm^{-1}**	**1529 cm^{-1}**
**PDI (L)**	1%	23%	/	3%	<0.5%	14%
**PDI (R)**	10%	1%	8%	5%	13%	/
**DDP (L)**	3%	8%	<0.5%	29%	<0.5%	83%
**DPP (R)**	18%	<0.5%	5%	30%	82%	1%
**BDT (L)**	36%	67%	85%	28%	1%	2%
**BDT (R)**	32%	<0.5%	1%	6%	4%	/
**Polymer**	89%	76%	92%	92%	87%	86%
**PDIs**	11%	24%	8%	8%	13%	14%

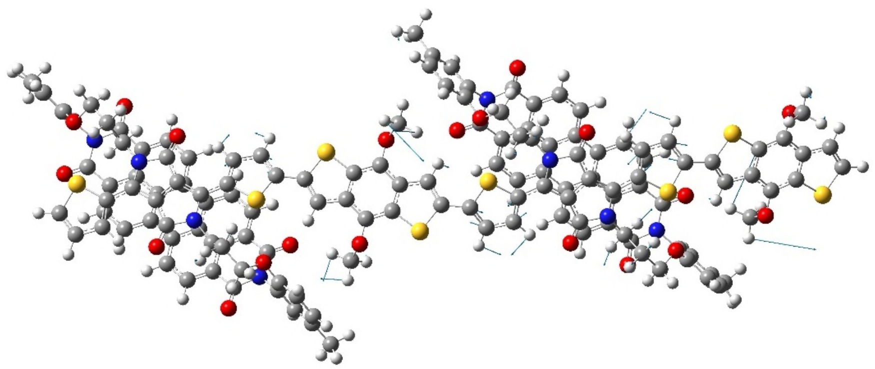

Supplementary Fig. 30 | Normal mode displacement of the TS-P3 dimer at 1440 cm^{-1}: Illustration of the vibrational mode showing atomic displacement vectors corresponding to the 1440 cm^{-1} Raman-active motion.

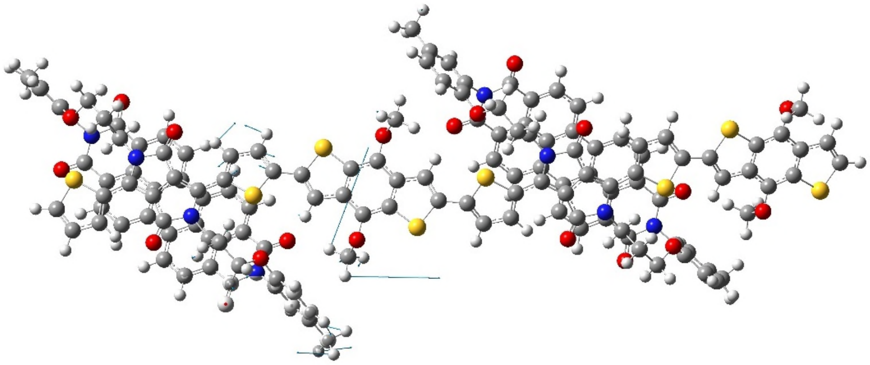

Supplementary Fig. 31 | Normal mode displacement of the TS-P3 dimer at 1441 cm^{-1}: Illustration of the vibrational mode showing atomic displacement vectors corresponding to the 1441 cm^{-1} Raman-active motion.

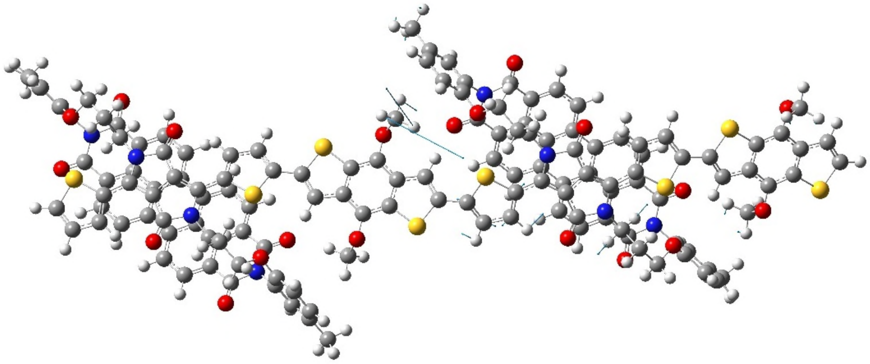

Supplementary Fig. 32 | Normal mode displacement of the TS-P3 dimer at 1444 cm^{-1}: Illustration of the vibrational mode showing atomic displacement vectors corresponding to the 1444 cm^{-1} Raman-active motion.

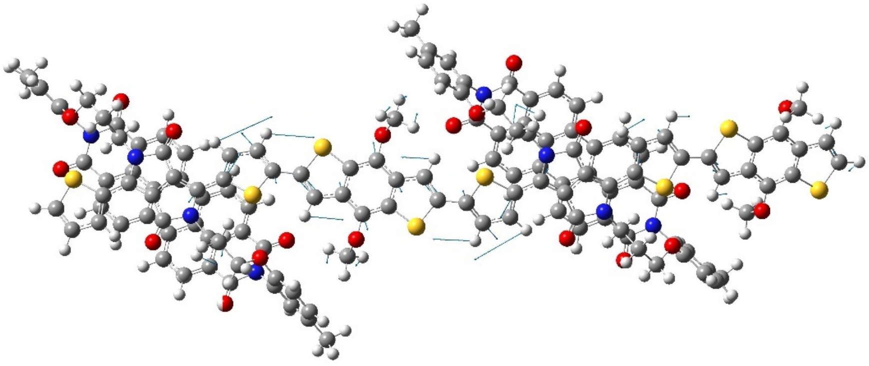

Supplementary Fig. 33 | Normal mode displacement of the TS-P3 dimer at 1495 cm⁻¹: Illustration of the vibrational mode showing atomic displacement vectors corresponding to the 1495 cm^{-1} Raman-active motion.

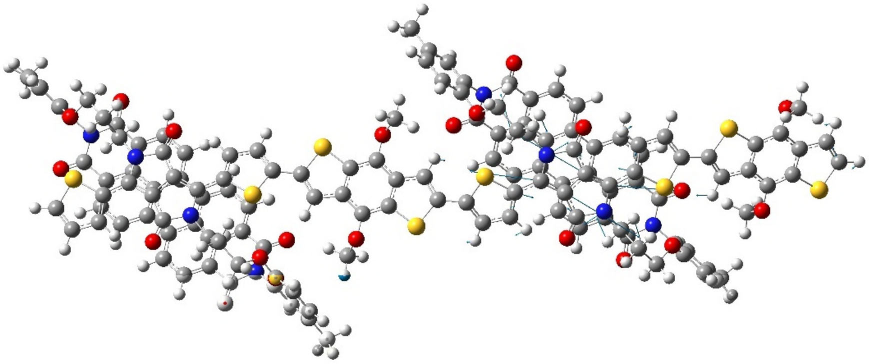

Supplementary Fig. 34 | Normal mode displacement of the TS-P3 dimer at 1527 cm^{-1}: Illustration of the vibrational mode showing atomic displacement vectors corresponding to the 1527 cm^{-1} Raman-active motion.

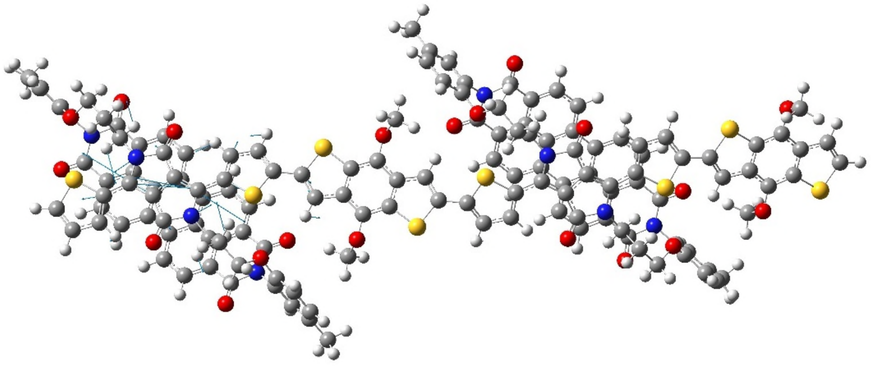

Supplementary Fig. 35 | Normal mode displacement of the TS-P3 dimer at 1529 cm^{-1}: Illustration of the vibrational mode showing atomic displacement vectors corresponding to the 1529 cm^{-1} Raman-active motion.

Time dependent (TD) DFT calculations were then performed for the optimized TS-P3 dimer structure (*i.e.*, at the Frank-Condon region) and for all the geometries obtained by displacing the structure (both in a positive and negative direction) along the relevant high-frequency normal modes. The displacements, expressed in Å, were performed by using the *GaussView 6* graphical interface. All the TD-DFT calculations were run by using the *screened* range-separated hybrid (SRSH) approach[20], where the LC-ωhPBE functional was used, along with the 6-311G(d,p) basis set. In this scheme, where the interelectron Coulomb operator is partitioned between a long- and a short-range domain, the range-separation parameter ω was optimally tuned (OT) in gas-phase (at $\omega = 0.103$ Bohr^{-1}) and the dielectric constant of toluene ($\varepsilon = 2.37$) was set *a-posteriori* via the adjustable parameter β, while the parameter α (that is the fraction of the Hartree-Fock exchange amount in the short-range domain) was kept fixed at 0.2, so to satisfy the relationship for which $1/_{\varepsilon} = \alpha + \beta$. All the (TD)-DFT calculations were carried out by using the Gaussian16 suite of packages[21].

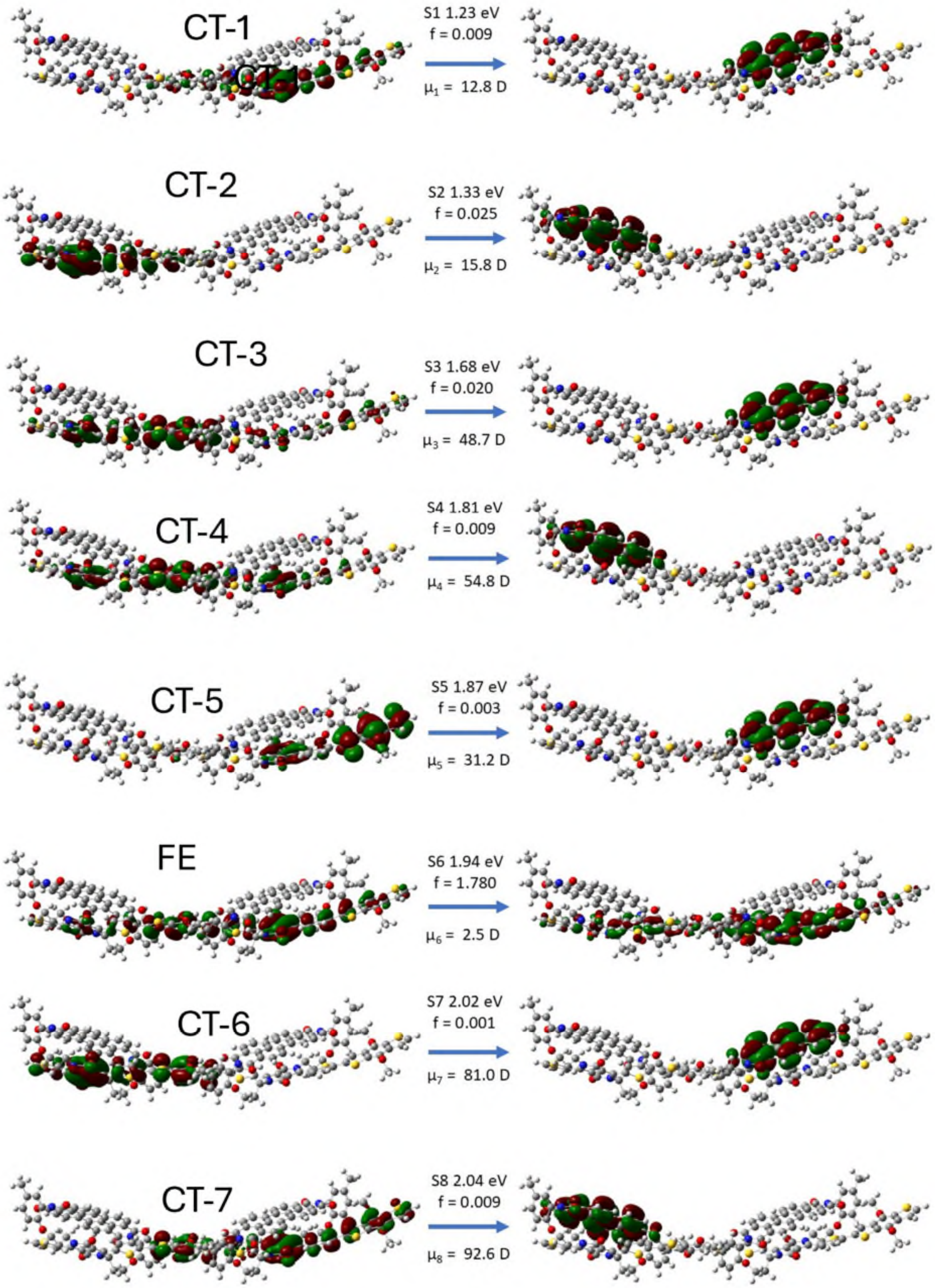

Supplementary Fig. 36 | Excitation energies, oscillator strengths, and dipole moments of the first eight excited states of the optimized TS-P3 dimer structure, along with the corresponding hole–particle natural transition orbitals.

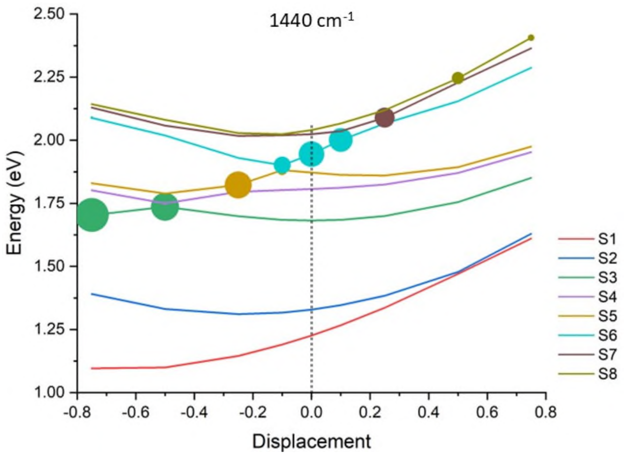

Supplementary Fig. 37 | Excited-state energy landscape of the TS-P3 dimer upon positive and negative displacements (in Å) along the normal mode at 1440 cm^{-1}: The dotted black line indicates the Frank-Condon region, while the size of the full circles over the different potential energy surfaces quantifies the oscillator strength of the bright Frenkel exciton state.

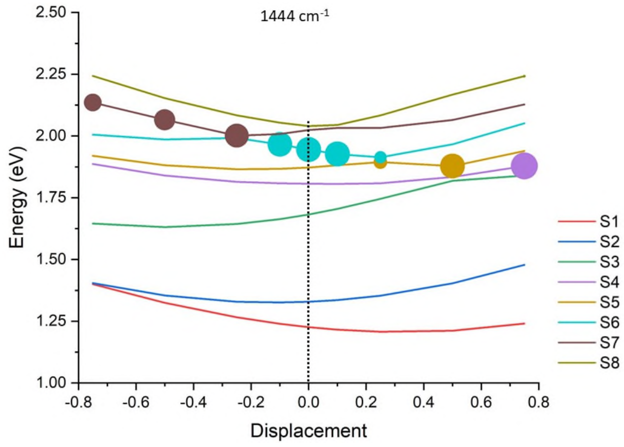

Supplementary Fig. 38 | Excited-state energy landscape of the TS-P3 dimer upon finite displacements (in Å) along the normal mode at 1444 cm^{-1}.

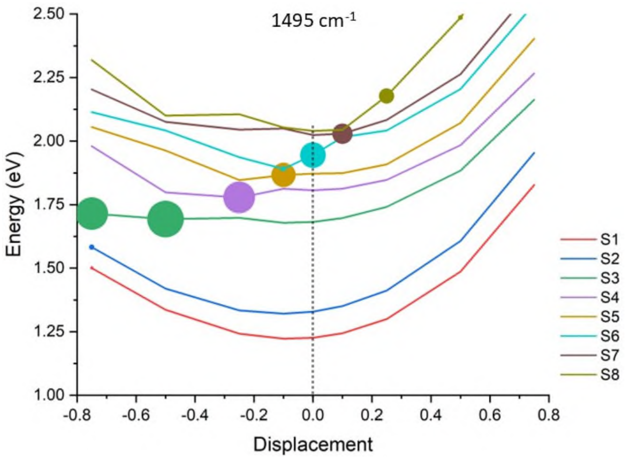

Supplementary Fig. 39 | Excited-state energy landscape of the TS-P3 dimer upon finite displacements (in Å) along the normal mode at 1495 cm^{-1}.

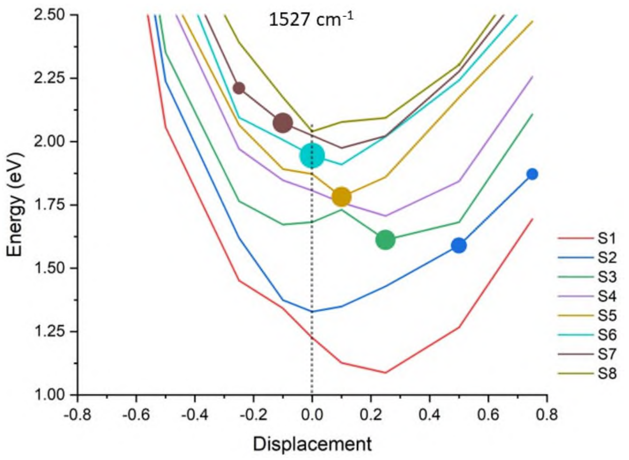

Supplementary Fig. 40 | Excited-state energy landscape of the TS-P3 dimer upon finite displacements (in Å) along the normal mode at 1527 cm^{-1}

Supplementary Note 16: Microscopic Origin of Charge Transfer

Because the PDI is connected to the polymer via a non-conjugated alkyl tether, charge transfer cannot proceed through a through-bond pathway but should occur via through-space interactions between the polymer donor backbone and the PDI acceptor. This design distinguishes our 'through space' constructs from other model heterojunctions, such as polymers with pendant donor/acceptor[22–25] groups or block copolymers[26–28], where through-bond coupling is significant.

Additional evidence for the 'structurally predefined through-space' nature of the transfer comes from a control experiment in which Ref-P and PDI were physically mixed at the same overall

composition. As shown in the supplementary figure 23, this mixture does not exhibit the characteristic PDI radical-anion signatures or accelerated polymer bleach decay observed in TS-P3. Instead, its kinetics closely resemble Ref-P alone, indicating negligible charge transfer. This confirms that random intermolecular encounters do not support efficient femtosecond CT, and that the covalent tether is essential for enforcing donor–acceptor ‘through-space’ proximity and dynamic CT-LE mixing through the vibrational modes.

To evaluate whether donor–acceptor coupling in TS-P3 is transmitted through the alkyl linkers or arises from through-space interactions, we conducted diabatization calculations on two variants of the model system: (a) the full TS-P3 dimer with all non-conjugated linkers included, and (b) a truncated structure with these linkers removed (supplementary fig. 49). The results of these quantum-chemical calculations are presented in SI Note 21, and the corresponding diabatic Hamiltonians are compiled in supplementary table S5. In both cases, the extracted electronic couplings are effectively identical, indicating that the linkers do not contribute to the coupling pathway. These findings confirm that the donor–acceptor interaction in TS-P3 is governed by through-space rather than through-bond coupling.

The difference between TS-P2 and TS-P3 therefore lies in the attachment site:

- TS-P3: PDI lies adjacent to the electron-deficient DPP moiety, enabling close co-facial geometry. Strong vibronic coupling to the ~1512 cm^{-1} which is a DPP-localised vibrational mode dynamically mix singlet (Frenkel exciton, FE) and CT states following photoexcitation, allowing for CT to be achieved on timescales much faster than would be expected when considering purely static electronic coupling interactions.
- TS-P2: PDI is tethered near the electron-rich BDT moiety, where spatial overlap and vibronic driving are weaker, leading to a slower ~376 fs transfer.

Together, these results show that through-space coupling is the operative mechanism in both systems, but vibronic enhancement at the DPP site makes CT in TS-P3 exceptionally fast.

Supplementary Note 17: Extraction of the ultrafast charge-transfer dynamics in model heterojunction

To further substantiate the sub-15 fs charge transfer (CT) dynamics inferred from the transient absorption spectra (Fig. 2e, main text), we compared the time-domain kinetics of TS-P3 with those of the reference polymer (Ref-P), as shown in Supplementary Fig. 41. Panel a shows the averaged $\Delta T/T$ traces of TS-P3 (blue) and Ref-P (orange) at 790–820 nm, where the acceptor (PDI) absorption dominates[29]. Both datasets exhibit identical coherent artefacts around time zero (< 20 fs), arising from cross-phase modulation and pump–probe overlap. Beyond this temporal region, however, the TS-P3 trace displays a pronounced rapid decay that is completely absent in Ref-P, indicating an additional ultrafast process linked to charge separation. It is also important to note that beyond ~20 fs there is no further spectral evolution in Ref-P (Fig. 2d), confirming that the contribution of coherent artefacts or cross-phase modulation is minimal after this time (vertical dotted line in Supplementary Fig. 41a). To isolate this CT-specific contribution, the Reference trace was subtracted from the TS-P3 signal. The resulting differential kinetics (Supplementary Fig. 41b) were analysed using a biexponential model comprising a dominant ultrafast component and a much weaker slower contribution. The fast component is attributed to electron transfer from the polymer backbone to the PDI acceptor, while the slower component likely reflects secondary processes following charge separation, such as dielectric or structural relaxation of the newly formed charge-separated state. This analysis yields a fast characteristic timescale of 18.1 ± 3.1 fs, corresponding to a conservative upper bound of 21.2 fs for the electron-transfer process. Importantly, this analysis supports the physical interpretation of the results: the electron-transfer process is faster than the period of the coherently generated vibrational mode (~26 fs), confirming that charge transfer occurs on a sub-vibrational timescale. The result implies that electron transfer in TS-P3 completes essentially within a single vibrational period of the 1512 cm^{-1} DPP-localised mode. In the following Supplementary Note 21, we corroborate these experimental observations with quantum-dynamical calculations which show the presence of a DPP-localized mode at approximately 1500 cm^{-1} strongly coupled with the electron transfer.

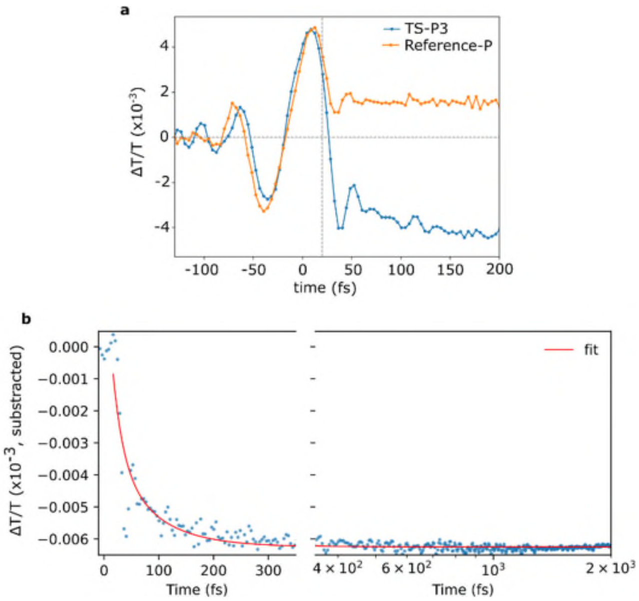

Supplementary Fig. 41 | (a) Transient absorption kinetics (ΔT/T) of TS-P3 (blue) and Ref-P (orange) averaged over 790–820 nm. The vertical dashed line marks time zero (< 20 fs), corresponding to the coherent artefact region. (b) Difference trace (TS-P3 – Ref-P) fitted with a dominant ultrafast decay component (red line), corresponding to an electron-transfer timescale of ~18 fs (upper bound ~21 fs).

Such near-instantaneous transfer is consistent with the vibronically coherent regime discussed in the main text and is further supported by the 100 fs-resolved transient absorption dynamics (Fig. 2b, main text), where slower sub-picosecond solvation relaxation is evident in Ref-P and TS-P2 but absent in TS-P3. Together, these findings confirm that the < 15 fs component represents the coherent electron transfer event rather than subsequent solvent or structural relaxation.

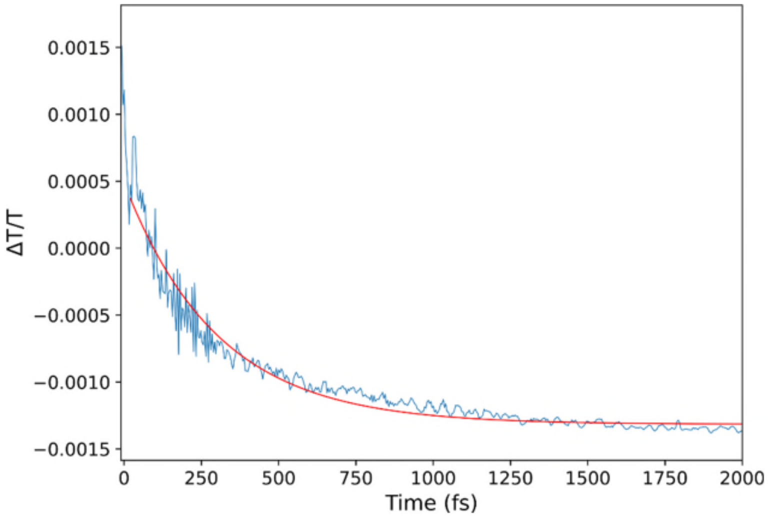

Supplementary Fig. 42 | Difference trace of TS-P2 after removal of the coherent artefact fitted with a mono-exponential decay (red line), giving a time constant τ = 376 fs.

Supplementary Note 18: Further discussion on the assignment of the 1283 cm^{-1} Vibrational Coherence in TS-P3

The assignment of the 1283 cm^{-1} vibrational coherence (VC) observed in TS-P3 requires careful consideration, as it lies close to the 1295 cm^{-1} Raman-active mode of isolated PDI. CW-Raman and off-resonant impulsive vibrational spectroscopy (IVS) of isolated PDI show a 1295 cm^{-1} mode (and a 1291 cm^{-1} coherence in IVS), both with symmetric lineshapes (Supplementary Fig. 43). By contrast, the 1283 cm^{-1} feature in TS-P3 is shifted, broadened, and asymmetric. The polymer backbone has no modes in the 1275–1325 cm^{-1} region, confirming that this feature originates from the PDI moiety. Below, we collate experimental, spectroscopic, and theoretical evidence to demonstrate that this feature arises from the PDI radical anion generated during ultrafast charge transfer (CT), rather than from a ground-state impulsive Raman (ISRS) response.

- The 1283 cm^{-1} coherence is tightly confined to the spectral region corresponding to the PDI radical-anion absorption (650–850 nm; Fig. 3j, main) and is absent from the neutral PDI bleach tail. This spectral localisation shows that the oscillation is resonantly detected through the excited-state charge-transfer (CT) absorption, rather than arising from ground-state Raman pathways. In particular, the pronounced deep-red localisation is inconsistent with ground-state ISRS, which would produce a broadband response extending across the neutral bleach.
- TS-P2, which contains the same PDI concentration as TS-P3, shows no 1283 cm^{-1} coherence, thereby ruling out any assignment to vibrational coherence generated by impulsive Raman scattering in the ground-state manifold. (Figure 3, main)
- A physical mixture of Ref-P and PDI with the same composition as TS-P3 likewise shows no 123 cm^{-1} coherence, and its kinetics closely match those of Ref-P alone. This further rules out an origin in vibrational coherence generated by impulsive Raman scattering in the ground-state manifold of TS-P3. (Supplementary Fig. 20)

To aid interpretation, we provide a decision-tree schematic (Supplementary Fig. 44) that summarises the combined evidence. Together, these results confirm that the 1283 cm^{-1} coherence in TS-P3 arises from the PDI radical anion on the excited-state CT surface, and not from ground-state ISRS.

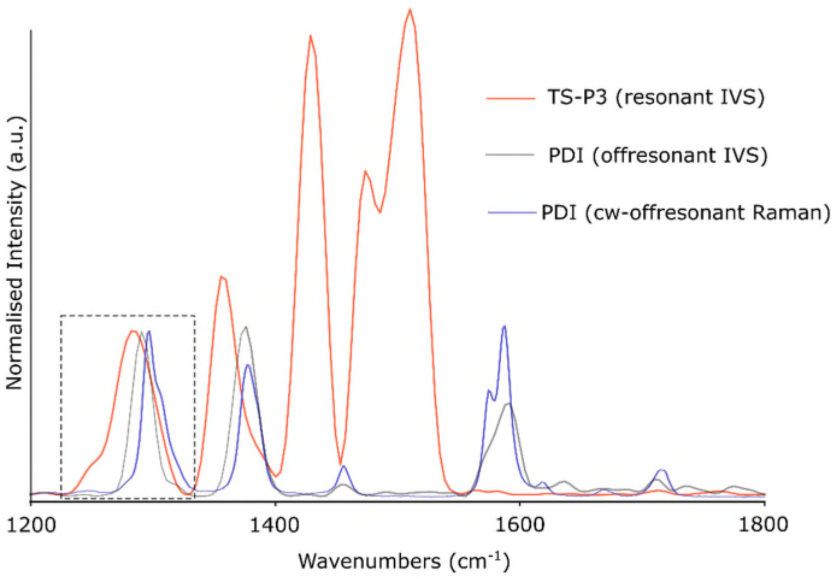

Supplementary Fig. 43 | Fourier transform spectra of vibrational coherences. Comparison of the resonant IVS spectrum of TS-P3 (orange) with cw-off-resonant Raman (blue) and off-resonant IVS (gray) spectra of isolated PDI. While the 1291–1295 cm^{-1} ground-state Raman/IVS modes of PDI exhibit symmetric lineshapes, the 1283 cm^{-1} feature in TS-P3 is distinctly shifted, broadened, and asymmetric. All Fourier transforms were performed over the same time window (40–1700 fs) to enable unbiased comparison.

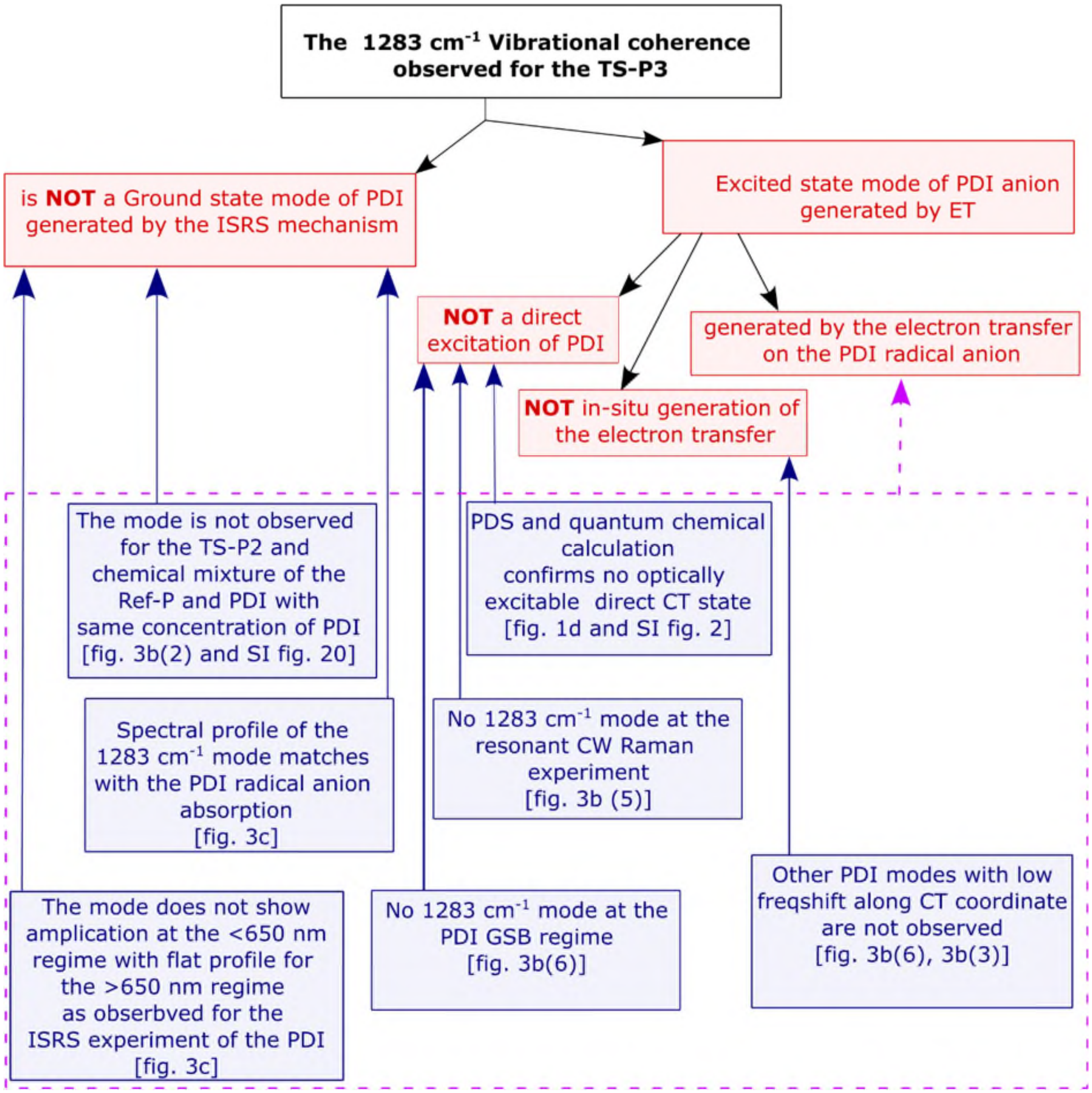

Supplementary Fig. 44 | Evidence for assignment of the 1283 cm^{-1} vibrational coherence in TS-P3. Decision tree summarising experimental and computational evidence for the microscopic origin of the 1283 cm^{-1} mode.

To quantify vibronic interactions in TS-P3, we computed the mode-specific gradients λ_k^{α} that define the linear vibronic coupling (LVC) Hamiltonian in Eq. S19 as explained more in detail in Supplementary Note 21, using the normal modes of isolated DPP–BDT and PDI fragments evaluated at the TS-P3 monomer geometry. Ground-state gradients were obtained from the harmonic QM Hessian, while excited-state gradients for the locally excited, cationic, and anionic states were constructed within the Vertical Gradient (VG) approximation (see Supplementary Note 21). This framework reveals that several high-frequency DPP–BDT modes exhibit large displacements, with the 1531 cm^{-1} mode acting as the principal driving coordinate in the

reduced model. The PDI modes, in contrast, begin to evolve only after the CT states are populated via this fast electron transfer, in agreement with the experimental observations (supplementary figure 51). This is especially clear for the PDI mode at 1299 cm^{-1}, whose frequency coincides with what has been found experimentally. In our impulsive vibrational spectroscopy measurements, the vibrational coherence along the 1283 cm^{-1} coordinate appears only after electron transfer, fully consistent with the simulations, which identify this mode as a PDI-anion vibration activated following CT-state formation.

Supplementary Note 19: Additional cw-Raman Analysis of PDI and TS-P3 Derivatives

To verify whether the 1283 cm^{-1} mode observed in the time-resolved measurements could originate from directly excitable PDI species (any *ground-state charge-transfer (CT) complex*), we performed comparative continuous-wave (cw) Raman spectroscopy using both 532 nm (resonant with PDI) and 785 nm excitation which should be resonant to any ground state CT complex if exists.

The spectra of TS-P3, TS-P3-5 %, and TS-P3-10 % (where PDI loading is reduced to 5 % and 10 %, respectively, please see Supplementary Fig. 7 for chemical structure) exhibit pronounced resonance-enhanced bands associated with the PDI chromophore (marked by red asterisks). Even at 5 % loading, the PDI modes strongly dominate over the polymer backbone features (black asterisks), confirming the strong Raman cross-section of PDI modes under resonant excitation. The strong enhancement of these bands at very low concentrations demonstrates the high sensitivity of the cw-Raman experiment to PDI vibrations when resonantly excited.

Under 785 nm excitation, TS-P3 spectra show markedly weaker overall Raman intensities, as expected for off-resonant conditions where the Raman scattering efficiency scales as $1 / \lambda^4$ (≈5× weaker at 785 nm than 532 nm). The signal-to-noise ratio is further reduced because of the fluorescence background. Importantly, no Raman feature is detected near 1283 cm^{-1} within the 3σ noise level, even in the TS-P3 spectrum (black trace, Fig. 45). The noise level was estimated from the flat baseline region (1700–2000 cm^{-1}), confirming no significant band at 1283 cm^{-1} with an amplitude > 3σ.

The combination of (i) strong, clearly detectable PDI modes under resonant excitation even at 5 % PDI content and (ii) the complete absence of any detectable feature near 1283 cm^{-1} at 785 nm off-resonant excitation provides compelling evidence that no ground-state CT complex or

directly excitable PDI species exists with an appreciable cross-section near 785 nm. If such a complex were present, the 1283 cm⁻¹ mode would be expected to under these conditions.

These results therefore confirm that the 1283 cm⁻¹ vibrational coherence observed in the transient absorption experiments originates not direct excitation of the PDI involved CT complex.

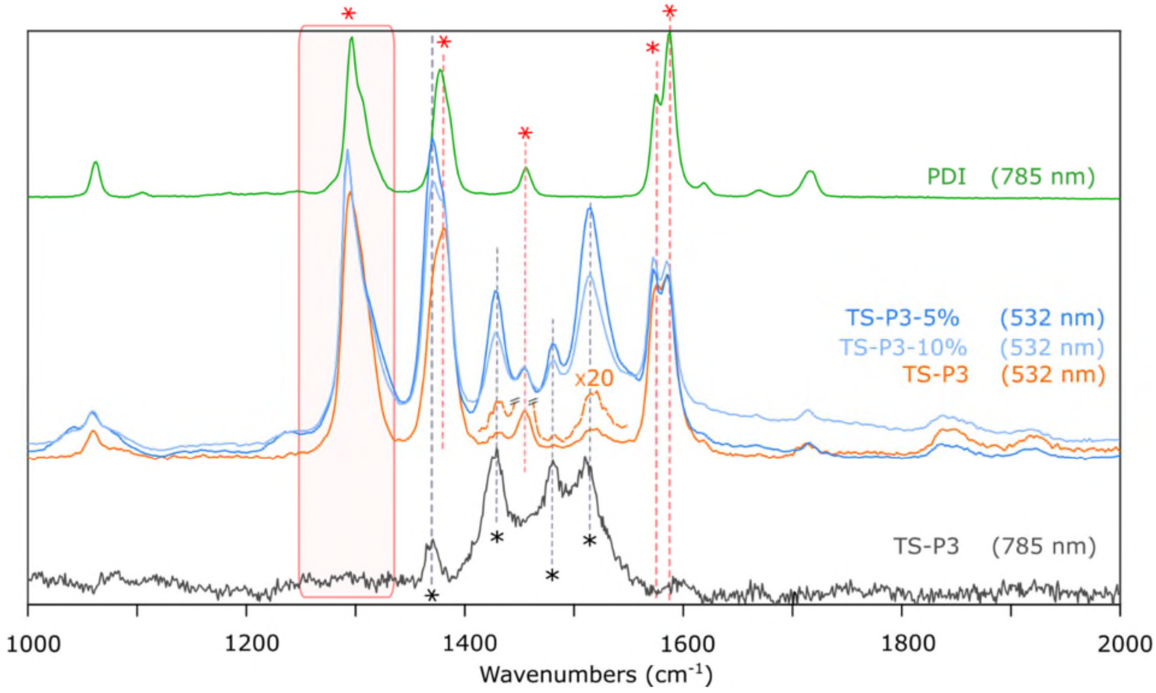

Supplementary Figure 45. cw-Raman spectra of PDI and TS-P3 under 532 nm and 785 nm excitation. Resonant 532 nm excitation (blue/orange) shows strong PDI modes (red asterisks), even at 5 % loading, while off-resonant 785 nm excitation (black) shows no detectable 1283 cm⁻¹ feature within the 3σ noise level (estimated from 1700–2000 cm⁻¹). This confirms the absence of any directly excitable ground-state CT complex.

Supplementary Note 20: Narrowband filtered inverse FFT and short time FFT (STFT) analysis

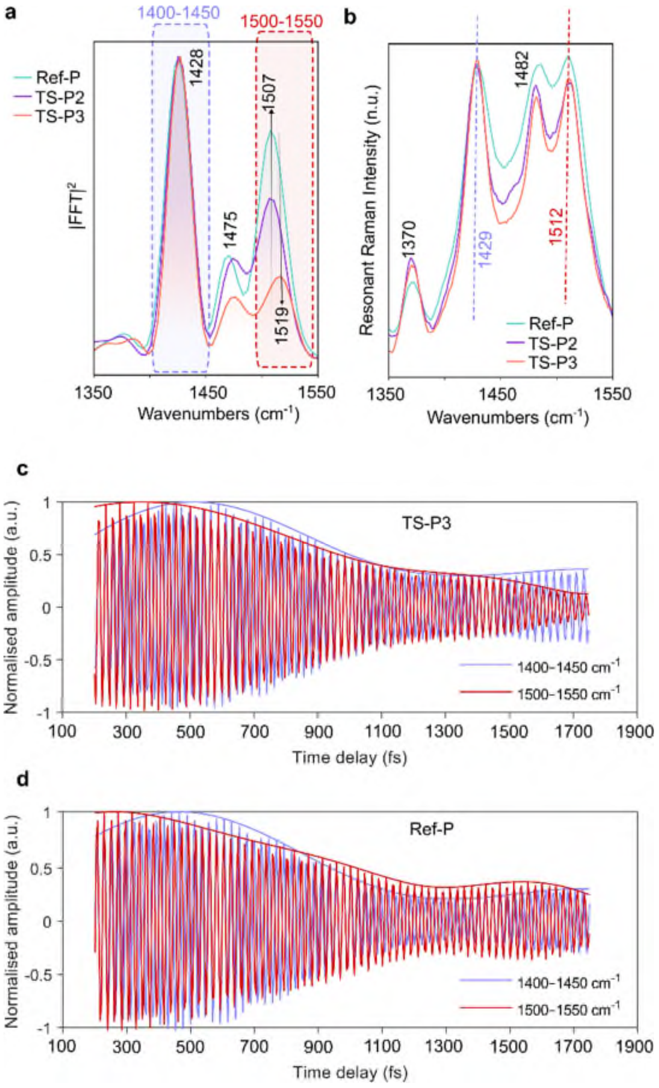

Supplementary Figure 46. Time-domain reconstruction of vibrational wave-packet oscillations by inverse Fourier filtering. a, CW-Resonant Raman spectra of the Ref-P, TS-P2, TS-P3 at the high frequency regime (excitation wavelength, 785 nm). **b**, Integrated Fourier transform IVS spectra for the probe wavelength range, λ = 890-920 nm which corresponds to the polymer backbone chromophore S1→Sn photo-induced absorption **c,d,** The transient absorption signal in the 890–930 nm probe range was first detrended using a 200 fs moving-average window to isolate coherent residuals for TS-P3(c) and Ref-P(d). The residual trace was then narrow-band filtered in the frequency domain within two wavenumber windows (1400–1450 cm^{-1} and 1500–1550 cm^{-1}) by zeroing all other spectral components and performing an inverse fast Fourier transform (iFFT). This procedure retrieves the oscillatory components associated with individual vibrational modes, while the analytic-signal (Hilbert transform) envelope provides their temporal dephasing behaviour. The faster decay of the 1500–1550 cm^{-1} component compared with the 1400–1450 cm^{-1} band indicates a shorter coherence lifetime of the mode driving electron transfer in TS-P3.

We analysed the photoinduced absorption region (890–920 nm) corresponding to the $S_1 \rightarrow S_n$ transition in the polymer, as presented in the main text. As shown in Figure 3k (main figure) and reproduced in Supplementary Figure 46a, a significant reduction in the FFT amplitude of the 1507 cm^{-1} mode is observed for TS-P3 compared with TS-P2 and Ref-P. In contrast, the

CW-resonant Raman spectra of all three systems exhibit nearly identical vibrational features (Figure 3l, reproduced as Supplementary Figure 46b). This indicates that the loss of intensity in the 1507 cm^{-1} mode for TS-P3 originates from vibrational decoherence processes occurring within the excited-state manifold, consistent with previous observations in small-molecule systems in solution[30,31].

To further examine the time-domain behaviour of these modes, we applied a narrow-band frequency mask and retrieved the corresponding temporal profiles via inverse FFT, following the procedure described in Ref[32]. The resulting data are shown in Supplementary Figures 46c (TS-P3) and 46d (Ref-P). The dephasing times extracted for TS-P3 are 1182 fs in the 1400–1450 cm^{-1} region and 870 fs in the 1500–1550 cm^{-1} region, while for Ref-P the corresponding values are 1139 fs and 1041 fs, respectively. These results demonstrate that the 1512 cm^{-1} mode in TS-P3 exhibits a faster dephasing rate than in Ref-P, whereas the 1428 cm^{-1} spectator mode shows comparable dephasing times in both systems. This is consistent with the quantum chemical calculations reported in Fig. 4 of the main text. The accelerated decoherence of the 1512 cm^{-1} mode in TS-P3 supports its role as a driving mode in the electron-transfer process. Such rapid dephasing of a driving vibrational mode is often attributed to nuclear nesting effects. We further note that this mode likely possesses an even faster component, as the electron transfer occurs on a sub-15 fs timescale; however, the early-time dynamics are obscured by coherent artefacts and cross-phase modulation, which introduce high-frequency oscillatory contributions.

We performed a narrow-band time-domain analysis and a short-time Fourier transform (STFT[32]) following the approach in ref[32], using a 200 fs window with 20 fs overlap. The STFT spectrogram (Supplementary Fig.47) shows a clear temporal lag in the appearance of the 1283 cm^{-1} mode, confirming that it arises from charge-transfer dynamics rather than direct impulsive excitation. Complementary inverse FFT analysis of the 1400–1450 cm^{-1} and 1500–1550 cm^{-1} windows reveals faster dephasing of the 1512 cm^{-1} DPP-localised mode in TS-P3 ($\tau \approx 870$ fs) relative to Ref-P ($\tau \approx 1040$ fs), while the spectator 1428 cm^{-1} mode remains unchanged. These results reveal the distinct temporal evolution of vibrational coherences, showing the 1283 cm^{-1} mode as a product-state vibration generated upon charge transfer, and the analysis further support our assignment of the 1512 cm^{-1} mode as the driving coordinate for the ultrafast electron-transfer process.

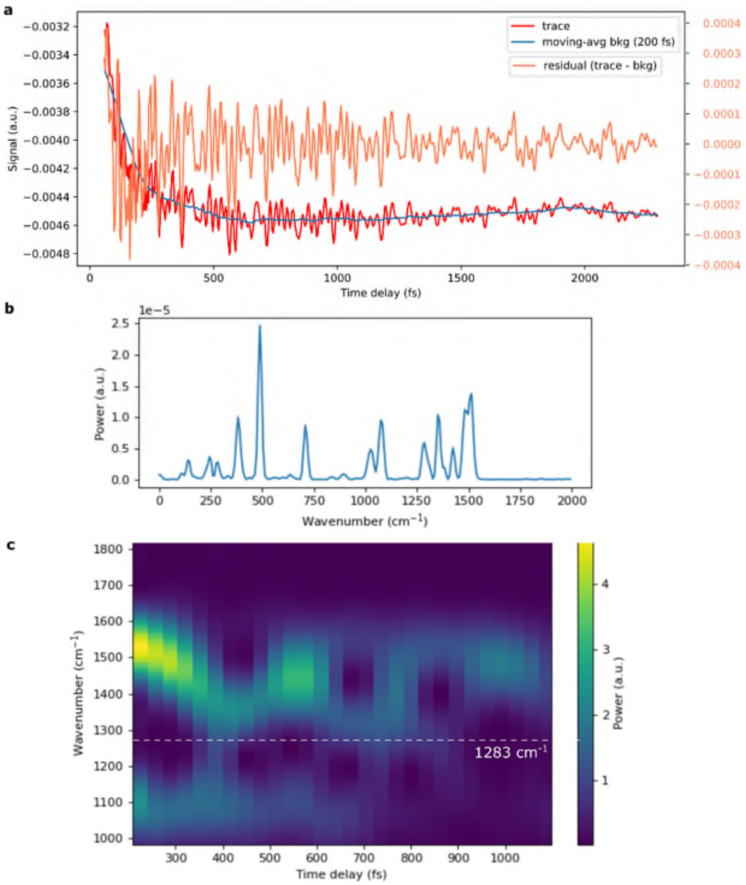

Supplementary Figure 47. Short-time FT (STFT) analysis of vibrational dynamics in TS-P3 at 890–930 nm probe window. (a) Transient absorption time trace (red) with its 200 fs moving-average background (blue) and the extracted residual oscillations (orange). The 200 fs moving average method is used for subtracting the background to remove the low-frequency component from the time domain data. The residual isolates coherent vibrational motion superimposed on the electronic response. (b) Fast Fourier transform (FFT) of the residual signal showing distinct vibrational modes between 200 and 1800 cm^{-1}. (c) Short-time Fourier transform (STFT) spectrogram revealing the temporal evolution of these modes. The dashed line marks the 1283 cm^{-1} feature, characteristic of the mode activated by electron-transfer dynamics. The colour scale represents oscillation power (a.u.).

Supplementary Note 21: Excited-state Quantum dynamics

Linear Vibronic Coupling Hamiltonian for TS-P3

The linear vibronic coupling Hamiltonian used to describe the ultrafast dynamics of the TS-P3 polymer is written as a sum of electronic, vibrational and electron-vibronic Hamiltonians:

$$\hat{H} = \hat{H}^{el} + \hat{H}^{vib} + \hat{H}^{el-vib} \qquad \text{(S16)}$$

The electronic Hamiltonian ($\hat{H}^{el}$) describes the various electronic excitations of the system in the diabatic representation. These include localized excitations (FE) on the DPP–BDT polymer backbone and charge-transfer (CT) excitations between the DPP–BDT polymer and the PDI unit. More specifically,

$$\hat{H}^{el} = \hat{H}_{\text{FE}} + \hat{H}_{\text{FE-CT}} \tag{S17}$$

Here, the diagonal elements correspond to the vertical energies of the diabatic excitations, while the off-diagonal elements, V_{kl}, represent the excitonic couplings that describe interactions between the FE and CT states included in the model. The electronic Hamiltonian is constructed using a diabatization procedure starting from the adiabatic states of the TS-P3 polymer, as detailed in the next paragraph.

The kinetic and potential components of the vibrational Hamiltonian $\hat{H}^{vib}$ are written in terms of the M (mass-weighted) dimensionless coordinates q_α and their conjugate momenta p_α of the modes of the TS-P3 system represented using:

$$\hat{H}^{vib} = \hat{K}^{vib} + \hat{V}^{vib} = \frac{1}{2}\sum_{\alpha} \omega_\alpha \, p_\alpha^2 + \frac{1}{2}\sum_{\alpha} \omega_\alpha \, q_\alpha^2 \tag{S18}$$

Where ω_α is the frequency of the specific hierarchically selected mode α (considering $\hbar = 1$). Note that a dimensionless coordinate q_α and related momenta q_α are found from the analogue quantity with dimensions by: $q_\alpha = \sqrt{\frac{\omega_\alpha}{\hbar}}\sqrt{m}x_\alpha$ and $p_\alpha = \frac{mv_\alpha}{\sqrt{m\omega\hbar}}$, respectively.

The electronic excitations, i.e., the formation of FE states and CT states, are strongly coupled to high-frequency vibrational modes as shown also by our impulsive vibrational spectroscopy experiments. Such electron-vibrational interactions are presented by the following Hamiltonian:

$$\hat{H}^{el-vib} = \hat{H}^{FE-vib} + \hat{H}^{CT-vib} \tag{S19}$$

The coupling between these quantized vibrational modes and the FE and CT excitations are incorporated into our Hamiltonian Eq. S16 by using a quantum displaced harmonic oscillator as explained the next paragraph and done by other groups.[33,34]

The first term represents the coupling between FE states (for instance those on the TS polymer) and a given mode α is:

$$\hat{H}^{FE-vib} = \sum_{k=1}^{N_{LE}} \sum_{\alpha}^{N_{exc}} \lambda_k^{\alpha,FE} q_\alpha \; |FE_k\rangle\langle FE_k| \tag{S20}$$

Similarly, the coupling between CT states and the nuclear degrees of freedom is given by:

$$\hat{H}^{CT-vib} = \sum_{k=1}^{N_{CT}} \sum_{\alpha}^{N_{cat}} \lambda_k^{\alpha,\mathrm{cat}} q_\alpha \; |CT_k\rangle\langle CT_k| + \sum_{k=1}^{N_{CT}} \sum_{\alpha}^{N_{ani}} \lambda_k^{\alpha,\mathrm{ani}} q_\alpha \; |CT_k\rangle\langle CT_k| \tag{S21}$$

$\lambda_k^{\alpha,(\mathrm{LE,ani,cat})}$ represent the gradients of the excited potential energy surface (excitonic, cationic or anionic states of the CT) at the equilibrium geometry of the ground state and are referred to as first-order intra-state electronic-vibrational coupling constants (which related also to the dimensionless shift g_k^α by $\lambda_k^{\alpha,} = g_k^\alpha \hbar\omega_\alpha$).[35] Note that the dimensionless shift is related to the Huang-Rhys factor by $S_k^\alpha = {g_k^\alpha}^2/2$. In Eq. S21, we make the assumption that the CT states are simply given by the formation of cationic state on the DPP-BDT polymer with and the anion on the PDI unit.

Diabatization and Electronic Hamiltonian

To carry out the quantum dynamics simulations, we first constructed the electronic Hamiltonian in Eq. S19 in the diabatic representation. This was achieved performing electronic structure excited state calculations on a reduced model comprising a single polymer (DPP-BDT) unit and a single PDI molecule. The use of this simplified model was motivated by computational efficiency and by the fact that the adiabatic states of this reduced system (Supplementary Fig.48) are representative of those in larger polymers (Supplementary Fig.36). This is also shown in supplementary table S3, with the most important difference being the number of states with CT character in the same energy window (which is higher for longer polymer systems).

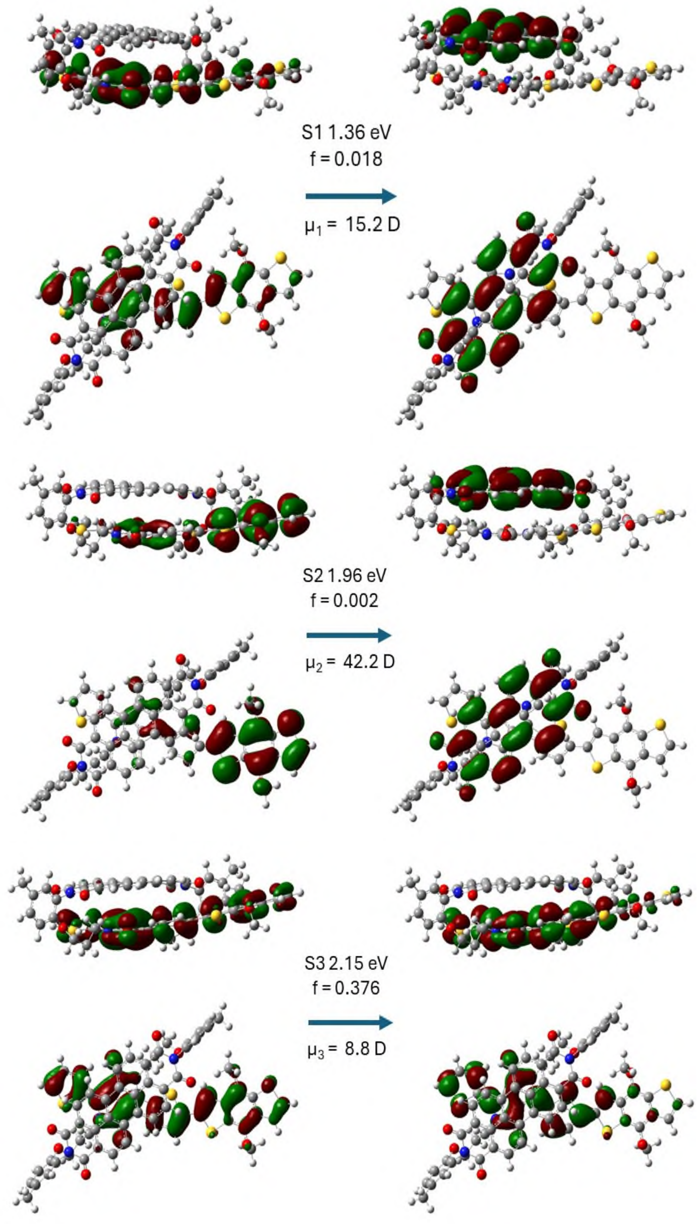

Supplementary Fig. 48 | Excitation energies, oscillator strengths, and dipole moments of the first three excited states of the optimized TS-P3 monomer structure, along with the corresponding hole–particle natural transition orbitals.

Supplementary Table S3 | Excitation energies (in eV) and their associated oscillator strengths for the TS-P3 dimer and monomer, as computed at the TD-DFT SRSH LC-ωhPBE/6-311G(d,p) level of theory.

TS-P3 dimer			TS-P3 monomer		
State (character)	Energy (eV)	Osc. Str.	State (character)	Energy (eV)	Osc. Str.
S1 (CT1)	1.23	0.009	S1 (CT1)	1.36	0.018
S2 (CT2)	1.33	0.025	S2 (CT2)	1.96	0.002
S3 (CT3)	1.68	0.020	S3 (FE)	2.15	0.376
S4 (CT4)	1.81	0.009			
S5 (CT5)	1.87	0.003			
S6 (FE)	1.94	1.776			

As shown in SupplementaryFig. 48 and Table S3, our TS-P3 monomer model, optimized at the ωB97X-D/6-31G(d,p) level of theory, features one bright excitation localized on the polymer and two lower-energy dark states corresponding to charge-transfer (CT) states from the polymer donor to the PDI acceptor. To evaluate the electronic couplings between the diabatic FE and intramolecular CT states, we employed an adiabatic-to-diabatic transformation using the multi-state fragment excitation difference–fragment charge difference (MS-FED-FCD) method. The MS-FED-FCD method extends and unifies the capabilities of previously developed property-based diabatization schemes, namely the two-state fragment excitation difference (FED)[36] and fragment charge difference (FCD)[37,38] approaches, which have been described in detail elsewhere[39–42]. The method partitions the system into two fragments, a donor and an acceptor (in our case the DPP-BDT unit and the PDI, respectively), and, using suitable additional operators, transforms the adiabatic Hamiltonian of the dimer (comprising two or more adiabatic states) into a diabatic representation yielding the electronic Hamiltonian in Eq. S17. This transformation enables direct evaluation of the excitonic couplings between FE and CT states from the diabatic Hamiltonian matrix.

Here, we applied the MS-FED-FCD diabatization in combination with TD-DFT at the LC-ωhPBE/6-311G(d,p) level of theory to both the monomer model TS-P3 system and the dimer TS-PX reported in the main text and Supplementary Note 15. Our simulations (see supplementary table S4) confirm that the electronic couplings between FE and CT states are small (i.e., 15-28 meV) in both cases, consistent with our experimental observations of weak excitonic effects influencing the optical absorption. Therefore, the ultrafast charge separation observed is not driven by strong excitonic couplings as it is the case for other system in the literature[43–45]. As discussed in the main text and shown by full quantum dynamics below, even in the presence of such weak electronic interactions, rapid charge separation occurs, driven by vibronic couplings and energy alignment between FE and CT states.

Supplementary Table S4 | Diabatic excitation energies (in eV) for the TS-P3 dimer and monomer, as computed with the multi-state fragment excitation difference–fragment charge difference (MS-FED-FCD) method on top of a TD-DFT SRSH LC-ωhPBE/6-311G(d,p) calculation. In the table there are also reported the electronic coupling (in meV) between the Frenkel exciton (FE) and the various CT states.

TS-P3 dimer			TS-P3 monomer		
	Energy (eV)	FE-CT coupling (meV)		Energy (eV)	FE-CT coupling (meV)
CT-1	1.23	15	CT-1	1.35	15
CT-2	1.33	-25	CT-2	1.96	28
CT-3	1.68	10	FE	2.20	/
CT-4	1.81	-1			
CT-5	1.87	3			
FE	1.96	/			

Supplementary Table S5: Diabatic Hamiltonian of the TP-P3 monomer with and without linkers. All the elements are in eV.

	TS-P3 mon.			TS-P3 mon. without linkers		
	LE	CT2	CT1	FE	CT2	CT1
FE	2.196	0.015	0.028	2.211	0.015	0.027
CT2	0.015	1.930	0.000	0.015	1.986	0.000
CT1	0.028	0.000	1.346	0.027	0.000	1.355

Our diabatic Hamiltonian shows that, as we move from low-energy charge-transfer (CT) states (e.g., CT1) to higher-energy CT states, the excitonic coupling decreases in both monomer and dimer polymers. This behaviour arises because the electron and hole become more spatially separated, as illustrated in Supplementary Fig.48 and S36. The larger separation leads to a weaker Coulomb attraction, which in turn results in a higher energy for the long-range CT states. We also note that the energy offset between FE and CT states in our simulations depends on the polymer size. Specifically, the energy offset decreases as the polymer length increase as we show in Table S4.

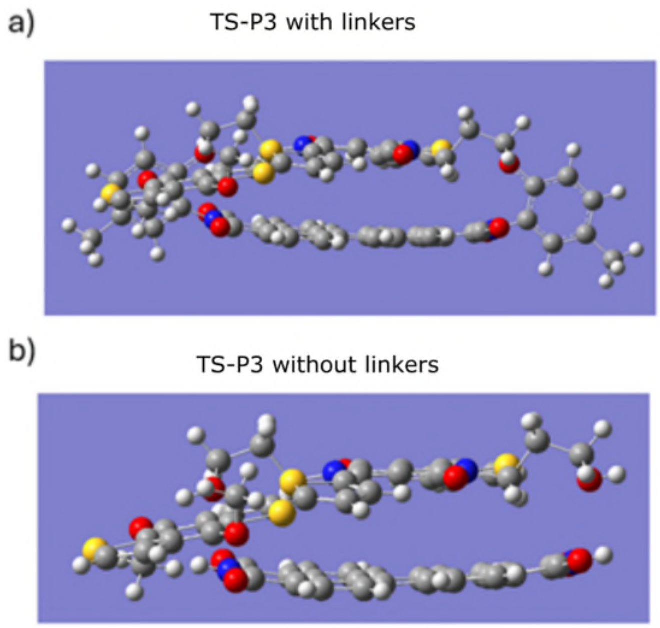

Supplementary Figure 49 | Representation of TS-P3 monomer with and without non-conjugated linkers connecting the DPP-BDT unit and the PDI.

.

As a further note, to understand whether the linkers connecting the DPP-BDT unit to the PDI affect the excitonic properties of the aggregate, we also performed the diabatization in the same TS-P3 model system where the non-conjugated linkers between donor and acceptor were removed (Supplementary Fig.49). The corresponding Hamiltonians are reported in supplementary table S5. Both cases yield similar electronic couplings, confirming that the non-conjugated linkers play a negligible role and that the interactions are primarily through-space in nature.

Normal mode analysis and Vibronic interactions

As observed in our experiments, the vibronic interactions play an important role in the ultrafast dynamics of the TS-P3 systems. To estimate the strength of these interactions, we computed the gradients $\lambda_k^{\alpha,(\mathrm{FE,ani,cat})}$ along the normal mode coordinates appearing in Eq. S20 and S21 using the normal modes of the isolated structures if DPP-BDT and PDI fragments taken at the optimized TS-P3 monomer geometry in its ground state in the gas phase (see Supplementary Fig. 48). These gradients, used to set-up the LVC Hamiltonian in Eq. S19, are computed analytically employing a (quantum) harmonic oscillator model. This approach allows us to compute the ground state potential energy surface (PES) in the harmonic approximation using the QM hessian and frequency at the isolated fragment geometries.

To build the excited-state PES, which in our case, refer to the locally excited state and the cationic state of the DPP-BDT molecule and the anionic state the PDI molecule, respectively, we adopt the Vertical Gradient (VG) approach as implemented in the FCclasses3.0 software.[46,47]. Within this approximation final-state PES is assumed to have the same normal modes and frequencies as the initial (ground) state potential. The VG accounts only for the effect of the dimensionless displacement g_α, for each mode α from the equilibrium position. These displacements are related to the gradients λ_k^α by: $\lambda_k^{\alpha,(\mathrm{FE,ani,cat})} = g_k^\alpha \hbar\omega_\alpha$ for a given k state. The frequencies are multiplied by a factor 0.949 as done for the TS-P3 dimer in Supplementary Fig.36.

We note that, in general, the gradients of different CT states are not identical. However, for simplicity, we assume here that all CT states have the same gradients, obtained by summing the gradients of the anionic and cationic contributions as shown in Eq. 21. This approximation could be relaxed by computing the gradient numerically for each CT state.[34,48] However, this is not practical, as it would require diabatizing the states for each normal mode of the TS-P3 monomer, which contains a large number of modes. For this reason, we dropped the index k in the estimate reported for the gradients Table S6. The relaxation energy of each mode can be estimated as well $E_\alpha^{rel} = \frac{{g^\alpha}^2}{2}\hbar\omega_\alpha$ and it is reported in the same Table S6.

These simulations clearly show that several high-frequency modes of the DPP-BDT are strongly coupled with both the exciton as well as the cationic states. In particular, the mode at 1531 cm^{-1} (at a frequency that agrees with the mode observed in IVS, 1519 cm^{-1}) shows the largest displacement and can be considered the driving mode of this reduced model. Concerning the PDI, our simulations show a strong coupling between the anion and the modes around ~1200-1300 cm^{-1}. This observation supports the fact that the vibrational coherence along the mode at 1283 cm^{-1} is generated upon the electron transfer. As we discuss below, these modes are clearly involved in the excited state dynamics of the system which support the experimental observed in the impulsive vibrational spectroscopy.

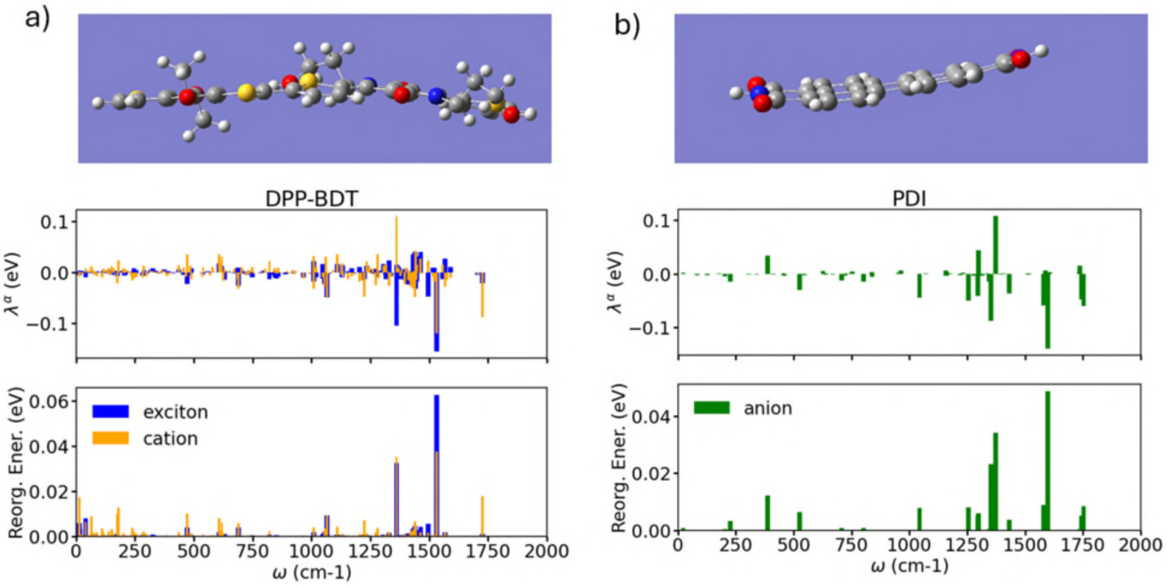

Supplementary Fig. 50 | Representation of the structures of a) DPP-BDT unit and b) PDI unit used to compute the gradients (middle panels) of the PES of exciton and cation of DPP-BDT and anion of PDI, respectively as well as related relaxation energies (bottom panels) as explained in the text.

Quantum dynamics

Nuclear wave-packet propagations were carried out using the MCTDH method[49–53], as implemented in the QUANTICS code.[54] The wavepackets were propagated for 200 fs in steps of 0.5 fs. As the number of modes is sizable, we relied on Multi-layer (ML) extension of MCTDH.[53] We adopted the variable mean field scheme with a Runge-Kutta integrator of order 5 and an accuracy threshold of 10^{-7}. Different numbers of single-particle functions (SPFs) for each layer as well as a different number of modes were tested for convergence of the results.

Notably, considering all normal modes for each diabatic state of TS-P3 monomer is still too demanding. To reduce the number of modes included in the Hamiltonian (Eq. S16), we selected a total of 36 most important modes (18 for the DPP-BDT and 18 for the PDI), with the largest contribution to the total relaxation energy. The selected modes and their corresponding gradients are reported in Table S6.

Table S6: Electron vibrational couplings (λ^{α}) and related relaxation energies (E_{α}^{rel}) of the 36 modes included in the LVC Hamiltonian for DPP-BDT excitonic and cation state as well as the PDI anionic state

DPP-BDT					PDI		
	Exciton		cation			anion	
ω_{α} (cm^1)	λ^{α} (eV)	E_{α}^{rel} (eV)	λ^{α} (eV)	E_{α}^{rel} (eV)	ω_{α} (cm^1)	λ^{α} (eV)	E_{α}^{rel} (eV)
1531.1	0.1543	0.0627	0.1197	0.0378	1598.8	0.1388	0.0486
1361.5	0.1050	0.0327	-0.1092	0.0353	1373.0	-0.1077	0.0341
1726.0	0.0214	0.0011	0.0876	0.0179	1353.5	0.0882	0.0232
14.0	0.0045	0.0059	0.0078	0.0173	387.5	-0.0342	0.0122
179.5	-0.0023	0.0001	-0.0240	0.0129	1579.4	0.0595	0.0090
472.3	0.0222	0.0042	-0.0347	0.0103	1753.0	0.0606	0.0084
175.6	0.0074	0.0013	0.0210	0.0101	1255.2	0.0500	0.0080
1066.2	0.0496	0.0093	0.0502	0.0095	1044.2	0.0451	0.0079
64.7	-0.0013	0.0001	-0.0119	0.0089	526.5	0.0292	0.0065
40.5	0.0090	0.0081	-0.0081	0.0065	1298.1	-0.0439	0.0060
607.1	-0.0170	0.0019	-0.0347	0.0080	1743.0	0.0477	0.0053
1224.2	0.0125	0.0005	0.0467	0.0072	1296.7	0.0407	0.0052
1438.9	-0.0401	0.0045	0.0487	0.0066	1431.3	0.0361	0.0037
619.1	-0.0134	0.0012	-0.0309	0.0062	226.9	0.0136	0.0033
690.4	0.0261	0.0040	0.0317	0.0059	802.9	0.0138	0.0010
1496.2	0.0464	0.0058	-0.0240	0.0015	707.0	0.0120	0.0008
1443.2	0.0323	0.0029	-0.0417	0.0049	22.6	-0.0021	0.0008
1109.5	-0.0159	0.0009	-0.0350	0.0045	1736.9	-0.0157	0.0006

The results of our quantum dynamics (QD) trajectories are reported in Supplementary Fig. 51. The first important observation is that a fast population transfer is recovered when using the Hamiltonian defined in supplementary table 5 with and energy off-set between the FE state and the CT1 and CT2 of 0.850 eV and CT2 and CT1 of 0.583 eV, respectively as found for our model TS-P3 monomer system (see yellow line in Supplementary Fig.51). However, this transfer remains slower than that observed experimentally and an important reason for that is that as discussed and shown before in Table S4, longer polymers show a reduced offset between FE and CT sates. Thus, we tested the sensitivity of the electron transfer with respect to such an offset, by decreasing the energy difference between FE -CT1 and CT2-CT1 linearly by factor 2. We find that the rate of this ultrafast transfer is strongly dependent on such an energy offset between the FE and CT states. The transfer gets faster when it becomes barrierless.

We note that when using an offset value comparable to the experimental estimate (around 200 meV), the transfer rate decreases to ~75fs, thus closing the gap with the experimental measurements. Remaining discrepancies might be attributed to the approximation of using the same

gradients for different CT states, the reduced number of nuclear degrees of freedom and the absence of non-local electron-vibrational couplings.

Despite these differences, our simulations clearly show the rapid generation of CT states that get populated on an ultrafast time scale. More importantly, they reveal that the fast-driving mode of the DPP-BDT polymer at 1531 cm^{-1}, together with contribution from the other vibrational modes, drives the initial ultrafast transfer. The PDI modes, in contrast, begin to evolve only after the CT states are populated via this fast electron transfer, in agreement with the experimental observations. This is especially clear for the PDI mode at 1299 cm^{-1}, whose frequency coincides with what has been found experimentally. In our impulsive vibrational spectroscopy measurements, the vibrational coherence along the 1283 cm^{-1} coordinate appears only after electron transfer, fully consistent with the simulations, which identify this mode as a PDI-anion vibration activated following CT-state formation.

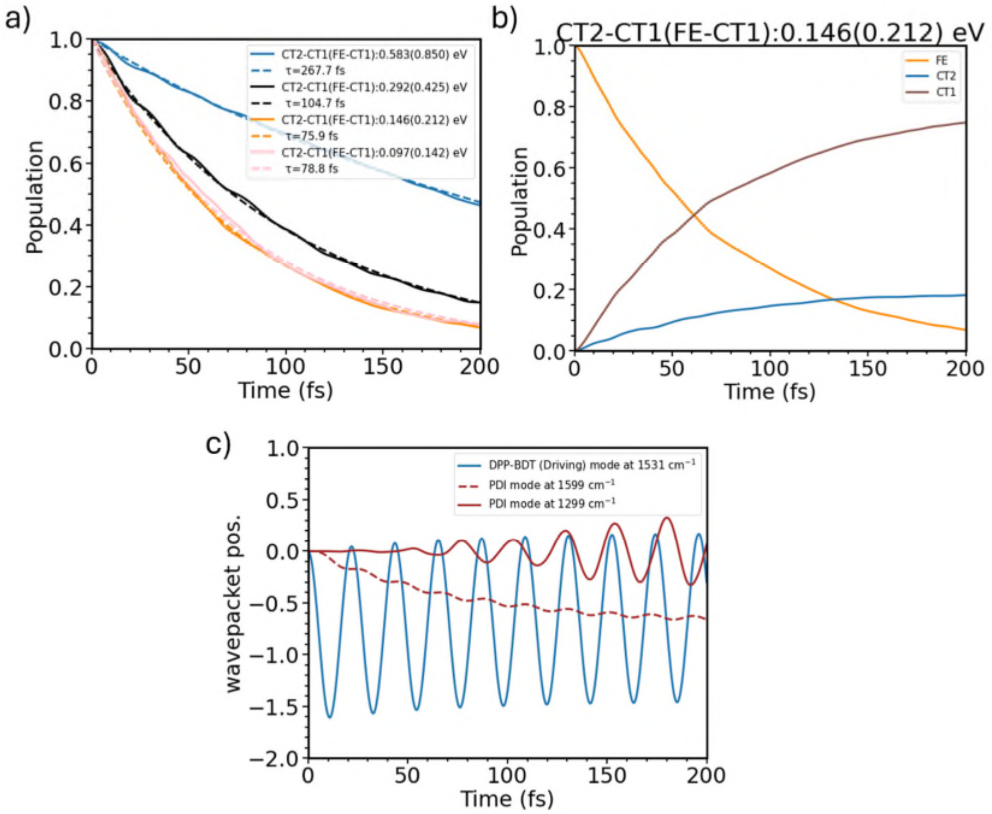

Supplementary Fig. 51: a) Population decay of the FE state and its fitting with a monoexponential function to extract the characteristic time scale of population transfer for different energy offsets with respect to CT1. Offsets are defined as the difference between FE and CT1 energies in parentheses, and CT2 and CT1 energies. b) Excited-state population for an energy offset of approximately 200 meV between the FE and CT states, as observed experimentally. c) Average wavepacket position as a function of time. This shows that the high-frequency DPP-BDT mode begins oscillating immediately with a period of ~20 fs, whereas the PDI modes (particularly the one at 1299 cm^{-1}) start oscillating only after the population transfer has occurred.

Supplementary References

1. Bässler, H., Kroh, D., Schauer, F., Nádaždy, V. & Köhler, A. Mapping the Density of States Distribution of Organic Semiconductors by Employing Energy Resolved–Electrochemical Impedance Spectroscopy. *Adv Funct Mater* **31**, (2021).

2. Royakkers, J. *et al.* Synthesis of model heterojunction interfaces reveals molecular-configuration-dependent photoinduced charge transfer. *Nat Chem* **16**, 1453–1461 (2024).

3. Falke, S. M. *et al.* Coherent ultrafast charge transfer in an organic photovoltaic blend. *Science (1979)* **344**, 1001–1005 (2014).

4. Matheson, A. B., Ruseckas, A., Pearson, S. J. & Samuel, I. D. W. Hole delocalization as a driving force for charge pair dissociation in organic photovoltaics. *Mater Horiz* **6**, 1050–1056 (2019).

5. Tang, J. *et al.* Achieving Efficient p-Type Organic Thermoelectrics by Modulation of Acceptor Unit in Photovoltaic π-Conjugated Copolymers. *Advanced Science* **9**, (2022).

6. Kovalenko, S. A., Dobryakov, A. L., Ruthmann, J. & Ernsting, N. P. Femtosecond spectroscopy of condensed phases with chirped supercontinuum probing. *Phys Rev A (Coll Park)* **59**, 2369–2384 (1999).

7. Gu, Z. *et al.* Exponentially modified Gaussian relevance to the distributions of translocation events in Nanopore-based single molecule detection. *Chinese Chemical Letters* **25**, 1029–1032 (2014).

8. Zewail, A. H. Optical molecular dephasing: principles of and probings by coherent laser spectroscopy. *Acc Chem Res* **13**, 360–368 (1980).

9. Ehara, T. *et al.* Dynamic Excited-State Localization Induced by Jahn-Teller Distortion Observed by Coherent Vibrational Spectroscopy. *J Am Chem Soc* https://doi.org/10.1021/jacs.5c06020 (2025) doi:10.1021/jacs.5c06020.

10. Kuramochi, H. *et al.* Femtosecond Polarization Switching in the Crystal of a [CrCo] Dinuclear Complex. *Angewandte Chemie - International Edition* **59**, 15865–15869 (2020).

11. Yoneda, Y., Sotome, H., Mathew, R., Lakshmanna, Y. A. & Miyasaka, H. Non-condon Effect on Ultrafast Excited-State Intramolecular Proton Transfer. *Journal of Physical Chemistry A* **124**, 265–271 (2020).

12. Ghosh, P. *et al.* Decoupling excitons from high-frequency vibrations in organic molecules. *Nature* **629**, 355–362 (2024).

13. Ostroverkhova, O. Organic Optoelectronic Materials: Mechanisms and Applications. *Chemical Reviews* vol. 116 13279–13412 Preprint at https://doi.org/10.1021/acs.chemrev.6b00127 (2016).

14. Meskers, S. C. J., Janssen, R. A. J., Saes, B. W. H., Lutz, M. & Wienk, M. M. Tuning the optical characteristics of diketopyrrolopyrrole molecules in the solid state by alkyl side chains. *Journal of Physical Chemistry C* **124**, 25229–25238 (2020).

15. Wang, L. & Asher, S. A. Refractive-index matching avoids local field corrections and scattering bias in solid-state Na 2SO 4 ultraviolet Raman cross-section measurements. *Appl Spectrosc* **66**, 157–162 (2012).

16. Debnath, T. *et al.* Coherent vibrational dynamics reveals lattice anharmonicity in organic–inorganic halide perovskite nanocrystals. *Nat Commun* **12**, (2021).

17. Debnath, T. *et al.* Coherent vibrational dynamics reveals lattice anharmonicity in organic–inorganic halide perovskite nanocrystals. *Nat Commun* **12**, (2021).

18. Rafiq, S. & Scholes, G. D. From Fundamental Theories to Quantum Coherences in Electron Transfer. *Journal of the American Chemical Society* vol. 141 708–722 Preprint at https://doi.org/10.1021/jacs.8b09059 (2019).

19. Parada, G. A. *et al.* Concerted proton-electron transfer reactions in the Marcus inverted region. *Science (1979)* **364**, 471–475 (2019).

20. Zheng, Z., Egger, D. A., Brédas, J. L., Kronik, L. & Coropceanu, V. Effect of Solid-State Polarization on Charge-Transfer Excitations and Transport Levels at Organic Interfaces from a Screened Range-Separated Hybrid Functional. *Journal of Physical Chemistry Letters* **8**, 3277–3283 (2017).

21. Frisch, M. J. *et al.* Gaussian 16, Revision A.03.

22. Yang, J. *et al.* Isoindigo-Based Polymers with Small Effective Masses for High-Mobility Ambipolar Field-Effect Transistors. *Advanced Materials* **29**, (2017).

23. Liang, S. *et al.* Double-Cable Conjugated Polymers with Pendent Near-Infrared Electron Acceptors for Single-Component Organic Solar Cells. *Angewandte Chemie - International Edition* **61**, (2022).

24. Zhang, F. *et al.* Soluble polythiophenes with pendant fullerene groups as double cable materials for photodiodes. *Advanced Materials* **13**, 1871–1874 (2001).

25. Tan, ao *et al.* Synthesis and Photovoltaic Properties of a Donor-Acceptor Double-Cable Polythiophene with High Content of C 60 Pendant. https://doi.org/10.1021/ma070052 (2007) doi:10.1021/ma070052.

26. Feng, G. *et al.* Thermal-Driven Phase Separation of Double-Cable Polymers Enables Efficient Single-Component Organic Solar Cells. *Joule* **3**, 1765–1781 (2019).

27. Wu, Y. *et al.* A conjugated donor-acceptor block copolymer enables over 11% efficiency for single-component polymer solar cells. *Joule* **5**, 1800–1815 (2021).

28. He, Y. *et al.* Industrial viability of single-component organic solar cells. *Joule* vol. 6 1160–1171 Preprint at https://doi.org/10.1016/j.joule.2022.05.008 (2022).

29. Mayerhöffer, U., Gsänger, M., Stolte, M., Fimmel, B. & Würthner, F. Synthesis and molecular properties of acceptor-substituted squaraine dyes. *Chemistry - A European Journal* **19**, 218–232 (2013).

30. Rafiq, S., Fu, B., Kudisch, B. & Scholes, G. D. Interplay of vibrational wavepackets during an ultrafast electron transfer reaction. *Nat Chem* **13**, 70–76 (2021).

31. Yoneda, Y. *et al.* Vibrational Dephasing along the Reaction Coordinate of an Electron Transfer Reaction. *J Am Chem Soc* **143**, 14511–14522 (2021).

32. Hong, Y., Schlosser, F., Kim, W., Würthner, F. & Kim, D. Ultrafast Symmetry-Breaking Charge Separation in a Perylene Bisimide Dimer Enabled by Vibronic Coupling and Breakdown of Adiabaticity. *J Am Chem Soc* **144**, 15539–15548 (2022).

33. Popp, W., Brey, D., Binder, R. & Burghardt, I. Quantum Dynamics of Exciton Transport and Dissociation in Multichromophoric Systems. *Annual Review of Physical Chemistry* **72**, 591–616 (2021).

34. Segalina, A. *et al.* How the Interplay among Conformational Disorder, Solvation, Local, and Charge-Transfer Excitations Affects the Absorption Spectrum and Photoinduced Dynamics of Perylene Diimide Dimers: A Molecular Dynamics/Quantum Vibronic Approach. *Journal of Chemical Theory and Computation* **18**, 3718–3736 (2022).

35. Troisi, A. Charge transport in high mobility molecular semiconductors: classical models and new theories. *Chemical Society Reviews* **40**, 2347 (2011).

36. Hsu, C.-P., You, Z.-Q. & Chen, H.-C. Characterization of the Short-Range Couplings in Excitation Energy Transfer. *The Journal of Physical Chemistry C* **112**, 1204–1212 (2008).

37. Voityuk, A. A. Estimation of electronic coupling in π -stacked donor-bridge-acceptor systems: Correction of the two-state model. *Journal of Chemical Physics* **124**, 1–7 (2006).

38. Yang, C.-H. & Hsu, C.-P. A multi-state fragment charge difference approach for diabatic states in electron transfer: Extension and automation. *The Journal of Chemical Physics* **139**, 154104 (2013).

39. Cupellini, L., Corbella, M., Mennucci, B. & Curutchet, C. Electronic energy transfer in biomacromolecules. *WIREs Computational Molecular Science* **9**, 1–23 (2019).

40. Nottoli, M. *et al.* The role of charge-transfer states in the spectral tuning of antenna complexes of purple bacteria. *Photosynthesis Research* **137**, 215–226 (2018).

41. Tölle, J., Cupellini, L., Mennucci, B. & Neugebauer, J. Electronic couplings for photo-induced processes from subsystem time-dependent density-functional theory: The role of the diabatization. *Journal of Chemical Physics* **153**, 184113 (2020).

42. Giannini, S. *et al.* Exciton transport in molecular organic semiconductors boosted by transient quantum delocalization. *Nature Communications* **13**, 2755 (2022).

43. Hong, Y., Schlosser, F., Kim, W., Würthner, F. & Kim, D. Ultrafast Symmetry-Breaking Charge Separation in a Perylene Bisimide Dimer Enabled by Vibronic Coupling and Breakdown of Adiabaticity. *J Am Chem Soc* **144**, 15539–15548 (2022).

44. Lin, C., Kim, T., Schultz, J. D., Young, R. M. & Wasielewski, M. R. Accelerating symmetry-breaking charge separation in a perylenediimide trimer through a vibronically coherent dimer intermediate. *Nat Chem* **14**, 786–793 (2022).

45. Bartynski, A. N. *et al.* Symmetry-breaking charge transfer in a zinc chlorodipyrrin acceptor for high open circuit voltage organic photovoltaics. *J Am Chem Soc* **137**, 5397–5405 (2015).

46. Avila Ferrer, F. J. & Santoro, F. Comparison of vertical and adiabatic harmonic approaches for the calculation of the vibrational structure of electronic spectra. *Physical Chemistry Chemical Physics* **14**, 13549 (2012).

47. Cerezo, J. & Santoro, F. FCclasses3 : Vibrationally-resolved spectra simulated at the edge of the harmonic approximation. *Journal of Computational Chemistry* **44**, 626–643 (2023).

48. Aarabi, M. *et al.* Quantum-Classical Protocol for Efficient Characterization of Absorption Lineshape and Fluorescence Quenching upon Aggregation: The Case of Zinc Phthalocyanine Dyes. *Journal of Chemical Theory and Computation* https://doi.org/10.1021/acs.jctc.3c00446 (2023) doi:10.1021/acs.jctc.3c00446.

49. Meyer, H. D. Studying molecular quantum dynamics with the multiconfiguration time-dependent Hartree method. *Wiley Interdisciplinary Reviews: Computational Molecular Science* **2**, 351–374 (2012).

50. Beck, M. The multiconfiguration time-dependent Hartree (MCTDH) method: a highly efficient algorithm for propagating wavepackets. *Physics Reports* **324**, 1–105 (2000).

51. Vendrell, O. & Meyer, H. D. Multilayer multiconfiguration time-dependent Hartree method: Implementation and applications to a Henon-Heiles Hamiltonian and to pyrazine. *Journal of Chemical Physics* **134**, (2011).

52. Meyer, H. D. & Worth, G. A. Quantum molecular dynamics: Propagating wavepackets and density operators using the multiconfiguration time-dependent Hartree method. *Theoretical Chemistry Accounts* **109**, 251–267 (2003).

53. Manthe, U. A multilayer multiconfigurational time-dependent Hartree approach for quantum dynamics on general potential energy surfaces. *The Journal of Chemical Physics* **128**, (2008).

54. Worth, G. A. Quantics: A general purpose package for Quantum molecular dynamics simulations. *Computer Physics Communications* **248**, 107040 (2020).
